# Expression profiling of microRNAs and isomiRs in conventional central chondrosarcoma

**DOI:** 10.1038/s41420-020-0282-3

**Published:** 2020-06-10

**Authors:** Antonina Parafioriti, Ingrid Cifola, Clarissa Gissi, Eva Pinatel, Laura Vilardo, Elisabetta Armiraglio, Andrea Di Bernardo, Primo Andrea Daolio, Armando Felsani, Igea D’Agnano, Anna Concetta Berardi

**Affiliations:** 1 Pathology Department, Azienda Socio Sanitaria Territoriale Gaetano Pini, Milan, Italy; 2 Institute for Biomedical Technologies (ITB), CNR, Segrate, Italy; 3 U.O.C. of Immunohaematology and Transfusion Medicine, Laboratory of Stem Cells, Spirito Santo Hospital, Pescara, Italy; 4 C.O.O., Azienda Socio Sanitaria Territoriale Gaetano Pini, Milan, Italy; 5 Institute of Biochemistry and Cell Biology (IBBC), CNR, Monterotondo, Italy; 6 Genomnia Srl, Bresso, Italy

**Keywords:** Bone cancer, Diagnostic markers

## Abstract

Conventional central chondrosarcoma (CCC) is a malignant bone tumor that is characterized by the production of chondroid tissue. Since radiation therapy and chemotherapy have limited effects on CCC, treatment of most patients depends on surgical resection. This study aimed to identify the expression profiles of microRNAs (miRNAs) and isomiRs in CCC tissues to highlight their possible participation to the regulation of pathways critical for the formation and growth of this type of tumor. Our study analyzed miRNAs and isomiRs from Grade I (GI), Grade II (GII), and Grade III (GIII) histologically validated CCC tissue samples. While the different histological grades shared a similar expression profile for the top abundant miRNAs, we found several microRNAs and isomiRs showing a strong different modulation in GII + GIII vs GI grade samples and their involvement in tumor biology could be consistently hypothesized. We then in silico validated these differently expressed miRNAs in a larger chondrosarcoma public dataset and confirmed the expression trend for 17 out of 34 miRNAs. Our results clearly suggests that the contribution of miRNA deregulation, and their targeted pathways, to the progression of CCC could be relevant and strongly indicates that when studying miRNA deregulation in tumors, not only the canonical miRNAs, but the whole set of corresponding isomiRs should be taken in account. Improving understanding of the precise roles of miRNAs and isomiRs over the course of central chondrosarcoma progression could help identifying possible targets for precision medicine therapeutic intervention.

## Introduction

Chondrosarcomas are a heterogeneous group of malignant bone tumors that are characterized by the production of a cartilage matrix^1^. Chondrosarcoma is the second most frequent primary malignant bone tumor after osteosarcoma^2^. The vast majority (85%) are conventional central chondrosarcomas (CCCs), which occur mainly in adulthood/old age, from an intramedullary location and most frequently involve the bones of the trunk, the pelvis, femur or humerus. The site and the histological grade of the tumors, based on criteria such as the presence of matrix changes, high cellularity, nuclear atypia, binucleation, mitotic rate, and necrosis, are currently the main prognostic factors, and in particular, histological grade is the single most important predictor of local recurrence and metastasis. Well-differentiated tumors with poor cellularity (low grades), classified as Grade I, are only locally aggressive, have good prognosis after surgical resection (commonly intralesional curettage) with 5-year survival rates of 90%; while poorly differentiated tumors with high cellularity (high grades), classified as Grade II and III, are associated with high metastatic potential with 5-year survival of 53%^3–6^. Chondrosarcomas are unresponsive to chemotherapy and radiotherapy and, to date, surgery is the treatment of choice (in order to prevent recurrence and metastasis, wide en-bloc excision is the preferred surgical treatment in Grade II and III cases)^7^.

MicroRNAs (miRNAs) are a class of regulatory non-coding small RNA molecules that control many biological pathways at post-transcriptional level and are involved in cancer progression^8,9^. Regulatory miRNAs are able to repress their targets by an imperfect base pairing between the seed sequence at the miRNA 5′ end and the complementary sites in the 3′-untranslated region (3′-UTR) of the target messenger RNAs (mRNAs). MiRNAs modulate target gene expression through translational repression or mRNA cleavage^10^. It is estimated that miRNAs are responsible for the regulation of translation of about 30–60% of human genes^11^. Changes in miRNA expression are associated with tumor progression and metastases, and miRNAs have been proposed as biomarkers for diagnosis and prognosis of various cancer types. Moreover, several studies showed that a single miRNA locus may transcribe and process not only the canonical miRNA sequence, but also a cluster of multiple miRNA isoforms (isomiRs) with expression, length, and sequence heterogeneities^12,13^. It has been shown that the isomiR expression diverges across different tissues, cell types, and developmental stages^12^.

Defining the CCC miRNA landscape is highly important and may reveal diagnostic and therapeutic markers and/or targets suitable to improve current chondrosarcoma precision medicine treatments. The aim of the present study was to investigate the involvement of miRNAs and isomiRs in a small but accurately selected CCC cohort to increase the knowledge about the miRNAs and miRNA-regulated pathways contributing to tumorigenesis in this pathology.

## Results

### miRNA expression in CCC

**Fig. 1:**
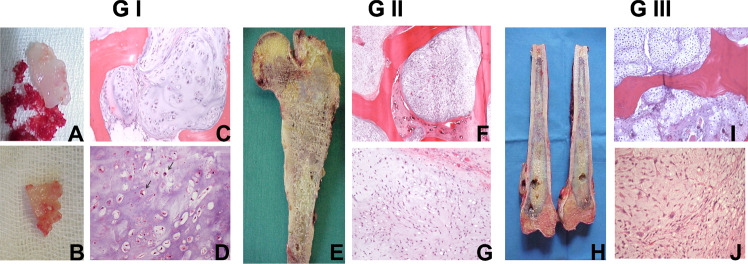
Multiple chondroid nodules from GI treated with curetage. **a**, **b** Macroscopic features; **c** microscopic features showing permeative growth patterns of GI CCC; **d** hypocellular cartilage and occasional binucleation (Arrow). GII CCC of proximal femoral shaft. **e** Macroscopic features on cut surface that show cartilaginous tumor with focal chalk-like gritty areas. **f**, **g** Microscopic features showing aggressive growth patterns of CCC with permeation of intertrabecular spaces within intramedullary cavity and intermediate power photomicrographs showing mixoid matrix and immature hypercellular cartilage. GIII CCC of femoral shaft. **h** Macroscopic features on cut surface. **i**, **j** Microscopic appearance of aggressive growth patterns with permeation of lamellar bone, showing marked increase in cellularity, nuclear atypia, binucleation, multinucleation, stromal myxoid changes, and spindling of chondrocytes.

Small RNA next-generation sequencing (NGS) was performed on a cohort of nine histologically validated CCCs (Fig. 1), comprising two Grade I (GI), three Grade II (GII), and four Grade III (GIII) cases (Table 1). It is well known that the clinical course of Grade I patients is diverse from Grade II and III^3,4,14^, thus, we decided to analyze our small cohort of patients comparing the GI cases to the GII and GIII together.

**Table. 1:** Clinical information of the patients analyzed.

Patient	ID	Location	Grade
1	10–777	Proximal humerus	WHO I
2	05–1568	Sacro	WHO I
3	06–1147	Proximal humers	WHO II
4	10–1374	Sacroiliac joint	WHO II
5	09–2231	Proximal fibula	WHO II
6	08–769	Proximal humerus	WHO III
7	04–356	Proximal humerus	WHO III
8	09–330	Proximal fibula	WHO III
9	09–608	Ischiopubic branch	WHO III

Age range 25–79, 4 female and 5 male.

Sequencing libraries were prepared from the small RNA fraction of the samples, size-selected to be enriched for miRNA fractions and then sequenced on a SOLiD 5500xl platform. After excluding low-quality reads and trimming adaptor sequences, the remaining reads were first mapped to the human genome (GRCh38/hg38) and then to miRbase (v21), to annotate known miRNAs in each sample. An average expression level of 0.001% was set as a threshold to exclude poorly expressed miRNAs. Using this threshold, we identified a total of 290 miRNAs expressed across all the CCC samples analyzed and reported in Supplementary Information (Table S1).

**Fig. 2:**
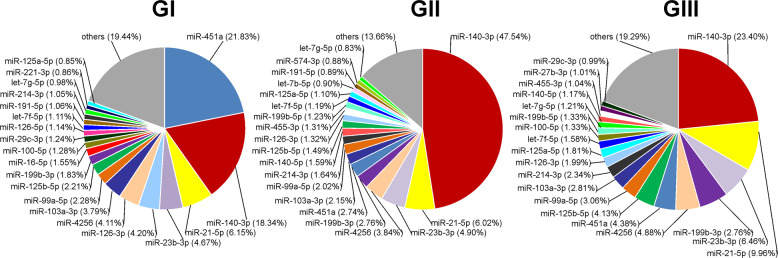
The 20 most abundant miRNAs expressed in Grade I, Grade II, and Grade III central chondrosarcomas. The colored sectors of the pie-charts identify each miRNA in Grade I (GI), Grade II (GII), and Grade III (GIII) CCC samples. The percentage of expression reported for each miRNA is calculated on the total miRNAs identified in each group.

Figure 2 shows the 20 most abundantly expressed miRNAs for GI, GII, and GIII samples, ordered according their abundance calculated as percentage of all the miRNAs expressed in each group. For all the three grades, these top 20 miRNAs represented over 80% of all the miRNA counts. Interestingly, we observed that most of the top 20 miRNAs were the same in all the three grades. Among them, miR-140-3p was the most abundant miRNA in GII (47.5%) and GIII (23.4%) groups and the second most abundant in GI samples (18.3%), where the most abundant miRNA was miR-451a (21.8%).

Nonetheless, when considering the globally most expressed miRNAs in the whole dataset, GII and GIII cases showed a more similar profile with respect to GI cases, which clustered apart as shown in Supplementary Information (Fig. S1).

Remarkably, when looking at miRNAs with a strong different expression level in GII + GIII vs GI cases of our collection (|log2FC|>2), we found 34 miRNAs, including 24 more- and 10 less-abundant in GII + GIII vs GI cases (even if only one -hsa-miR-489-3p- reaching statistical significance (FDR-BH < 0.1) due to small sample size) (Table 2). Among them, we noticed the presence of miR-451a, which we described above as one of the top 20 of all the three grades and the most abundant miRNA in GI group. Here, with a log2FC of −2.6, it was one of the miRNAs whose expression was most reduced in GII + GIII vs GI samples.

**Table 2:** List of miRNAs differently modulated in GII + GIII vs GI CCCs.

	Mean GI	Mean GII + GIII	Log2FC (GII + GIII) vs GI	*p*-Value	FDR-BH
*hsa-miR-206*	5.76	163.17	4.82	0.0452	0.56
*hsa-miR-31-3p*	3.71	47.59	3.68	0.0455	0.56
*hsa-miR-539-5p*	11.46	120.21	3.39	0.1733	0.80
*hsa-miR-382-5p*	42.82	392.61	3.20	0.2301	0.80
*hsa-miR-31-5p*	443.79	4027.37	3.18	0.0599	0.63
*hsa-miR-154-5p*	14.26	121.08	3.09	0.3019	0.80
*hsa-miR-335-5p*	69.42	563.24	3.02	0.0226	0.50
*hsa-miR-296-5p*	13.30	96.01	2.85	0.0039	0.19
*hsa-miR-337-5p*	29.08	202.89	2.80	0.1986	0.80
*hsa-miR-323b-3p*	13.86	95.72	2.79	0.2853	0.80
*hsa-miR-379-5p*	56.51	370.55	2.71	0.2880	0.80
*hsa-miR-133a-3p*	4.59	29.96	2.71	0.0210	0.50
*hsa-miR-487b-3p*	45.91	297.36	2.70	0.3172	0.80
*hsa-miR-127-3p*	34.01	214.65	2.66	0.3235	0.80
*hsa-miR-323a-3p*	20.00	121.45	2.60	0.3016	0.80
*hsa-miR-376a-5p*	19.63	116.84	2.57	0.3179	0.80
*hsa-miR-329-3p*	18.64	108.40	2.54	0.3207	0.80
*hsa-miR-134-5p*	65.72	367.31	2.48	0.4159	0.80
*hsa-miR-299-5p*	20.03	108.46	2.44	0.3034	0.80
*hsa-miR-337-3p*	55.00	284.17	2.37	0.3529	0.80
*hsa-miR-136-5p*	39.08	197.20	2.34	0.4154	0.80
*hsa-miR-409-3p*	112.71	545.22	2.27	0.3481	0.80
*hsa-miR-485-3p*	14.34	66.37	2.21	0.3792	0.80
*hsa-miR-369-5p*	12.70	52.10	2.04	0.4218	0.80
*hsa-miR-142-5p*	3066.08	762.90	−2.01	0.0277	0.50
*hsa-miR-378c*	1792.81	431.96	−2.05	0.0029	0.19
*hsa-miR-20b-5p*	397.40	89.68	−2.15	0.0391	0.52
*hsa-miR-4284*	142.31	30.78	−2.21	0.0801	0.72
*hsa-miR-144-3p*	1469.84	312.46	−2.23	0.0700	0.67
*hsa-miR-144-5p*	1064.99	219.75	−2.28	0.0322	0.51
*hsa-miR-106a-5p*	78.37	16.17	−2.28	0.0142	0.43
*hsa-miR-451a*	218299.38	36740.32	−2.57	0.0179	0.46
*hsa-miR-146a-5p*	503.56	76.59	−2.72	0.0039	0.19
*hsa-miR-489-3p*	495.25	27.07	−4.19	0.0004	0.05

Mean of the normalized counts (CPM) for each group and log2 fold-change (log2FC) are reported. *P*-values and false discovery rate (corrected by Benjamini–Hochberg method, FDR-BH) were calculated by EdgeR exact test method. Significant FDR < 0.1.

Using Diana Tools TarBase^15^, we determined the mRNAs targeted by these 34 strongly differently expressed miRNAs and analyzed the main pathways they are involved in using Reactome Pathways Database^16^. Table 3A, B displays the pathways enriched by the genes targeted by the miRNAs found more and less expressed, respectively, in our GII + GIII vs GI cases. Among the pathways targeted and, as consequence, probably inhibited by the more expressed miRNAs, we mainly found: (i) immune regulatory signaling, such as interferon signaling and ER-phagosome pathways; (ii) adhesion signaling, such as syndecan interactions; (iii) regulation of RUNX1 expression (Table 3A). Conversely, the less expressed miRNAs were able to target and control the signaling pathways of FGFR, ERK, and WNT (Table 3B).

**Table 3:** Pathways targeted by the miRNAs found (A) more expressed in GII + GIII vs GI cases and (B) less expressed in GII + GIII vs GI cases.

Pathway name	Entities found	Entities total	*p*-Value	FDR	Genes
A					
Antigen presentation: Folding, assembly and peptide loading of class I MHC	62	102	1.11E−16	3.06E−14	CALR, ERAP1, ERGIC2, HLA-A, HLA-B, MAP2, SAR1B, TWF1
Interferon signaling	77	250	1.11E−16	3.06E−14	CIITA, HLA-A, HLA-B, ICAM1, IFNG, IRF1, IRF2, IRF6, JAK1, PTPN1, SOCS3, SUMO1, TRIM2, TRIM29, KPNA5, KPNB1, NUP160, PLCG1, PPM1B
ER-phagosome pathway	59	165	1.11E−16	3.06E−14	CALR, HLA-A, HLA-B, SEC61B, SNAP25
Immunoregulatory interactions between a lymphoid and a non-lymphoid cell	62	316	5.89E−10	1.08E−07	COL1A1, HLA-A, HLA-B, ICAM1, PDCL, PRKAA2, SLAMF6, VKORC1L1
Apoptotic execution phase	13	54	6.41E−04	8.14E−02	CAD, CSRP1, KPNB1, MAP2, PAK2, PARP1, PPP2R2A, SATB1, SERP1, SH3BP2, SH3PXD2A, TJP1
Syndecan interactions	8	29	3.01E−03	3.32E−01	ACTN1, COL1A1, COL5A1, COL5A2, ITGA2, PRKAA2, SDC4, THBS1
RUNX1 regulates expression of components of tight junctions	4	8	4.00E−03	4.19E−01	CBFB, PPP2R2A, TJP1

B					
Neddylation	43	241	2.02E−04	1.22E−01	ANKRD52, ASB1, ATP6V1A, CAND1, CD2AP, CHD9, COPS2, CUL1, CUL3, CUL5, DCAF10, DCAF8, DCUN1D1, DHTKD1, DTL, EPAS1, FBXL3, FBXO30, FEM1B, FEM1C, GBF1, GLS, KBTBD6, KLHL20, KLHL42, LZIC, MED13, MSMO1, PRICKLE2, PSMF1, RBBP7, RBX1, RPN2, SOCS4, SOCS6, TCERG1, TULP4, UBE2Q2, UBXN7, VHL, VLDLR, XRN1
Cyclin D associated events in G1	14	48	3.90E−04	1.22E−01	CCND1, CCND2, CCNH, CDK6, CDKN1A, CDKN1B, CUL1, E2F3, RB1, RBL1, RBL2
FOXO-mediated transcription of cell cycle genes	10	27	4.33E−04	1.22E−01	CCNG2, CDKN1A, CDKN1B, RBL2, SMAD3, CCNG2, CDKN1A, CDKN1B, RBL2
Nuclear Receptor transcription pathway	20	86	4.80E−04	1.22E−01	ATL3, CCNI, CEP55, CIT, NR3C1, NR6A1, NRBP1, RORA
Amino acids regulate mTORC1	16	61	4.90E−04	1.22E−01	ATP6V0E1, ATP6V1A, ATP6V1C1, ATP6V1G1, FNIP2, GPD2, KLHL20, LAMTOR1, MIOS, MORF4L1, MYO1C, NLGN1, RPN2, RRAGD, SEC23A, SEH1L, SERINC1, VAPA
RUNX3 regulates WNT signaling	6	10	5.40E−04	1.22E−01	CCND1, CTNNB1, RUNX3, TCF4, TCF7L1, ZFYVE16
Signaling by PDGFRA extracellular domain mutants	8	19	7.04E−04	1.27E−01	KRAS, NRAS, PDGFRA, PIK3R1, SOS1, STAT1, STAT3
Antigen processing: Ubiquitination & proteasome degradation	50	315	8.83E−04	1.45E−01	AFF4, ANKRD52, ASB1, ATP6V1A, BIRC6, CD2AP, CDC23, CHD9, CUL1, CUL3, CUL5, DHTKD1, FBXL3, FBXO30, GBF1, GLS, HERC1, ITCH, KBTBD6, KDM6B, KLHL20, KLHL42, LNPEP, LZIC, MKRN1, MSN, MYLIP, PABPN1, PPTC7, PRICKLE2, PSMF1, RBX1, RLIM, RNF138, RNF19A, RPN2, SH3RF1, SIAH2, UBE2B, UBE2G1, UBE2J1, UBE2K, UBE2Q2, UBE2W, UBE2Z, UBE3C, UBE4A, VHL, WWP1
Signaling to ERKs	12	42	1.17E−03	1.76E−01	CRKL, CWC22, E2F3, FRS2, GM2A, KRAS, MAPK1, NRAS, RIT1, SNRNP27, SOS1, YWHAB
Signaling by FGFR3 fusions in cancer	7	17	1.69E−03	1.94E−01	FRS2, GAB1, KRAS, NRAS, PIK3R1, SOS1
Signaling by insulin receptor	20	97	1.97E−03	1.97E−01	ATP11B, ATP6V0E1, ATP6V1A, ATP6V1C1, ATP6V1G1, FRS2, GAB1, GPD2, KRAS, MAPK1, NRAS, PIK3C3, PIK3R1, RPN2, SERINC1, SOS1, THBS2, TNPO1, VAPA
Signaling by NTRKs	29	166	2.72E−03	2.45E−01	CRKL, CWC22, DHTKD1, E2F3, EP300, F3, FRS2, GAB1, GM2A, KRAS, MAPK1, MEF2A, NRAS, PIK3R1, PPP2CB, RAB11FIP5, REST, RHOA, RIT1, RPS6KA3, SLC30A7, SNRNP27, SOS1, STAT3, TRIB1, YTHDF1, YWHAB
TFAP2 (AP-2) family regulates transcription of cell cycle factors	4	6	3.15E−03	2.59E−01	CDKN1A, DHTKD1, KDM5B, TFAP2C
Downstream signaling of activated FGFR4	10	36	3.53E−03	2.76E−01	ATP11B, FRS2, GAB1, KRAS, NRAS, PIK3R1, SOS1, THBS2, TNPO1

### In silico miRNA validation in a chondrosarcoma public dataset

To validate the 34 miRNAs with a strong different expression level in our GII + GIII vs GI grade CCC samples, we analyzed the recently published miRNA sequencing data of a larger chondrosarcoma dataset including GI, GII, and GIII grade cases.

Globally, out of the 34 miRNAs strongly differently expressed in our samples, seven miRNAs resulted under the expression threshold (at low level of expression also in our case series) and thus not expressed in the public dataset (hsa-miR-136-5p, hsa-miR-144-3p, hsa-miR-144-5p, hsa-miR-31-3p, hsa-miR-376a-5p, hsa-miR-4284, hsa-miR-489-3p).

**Fig. 3:**
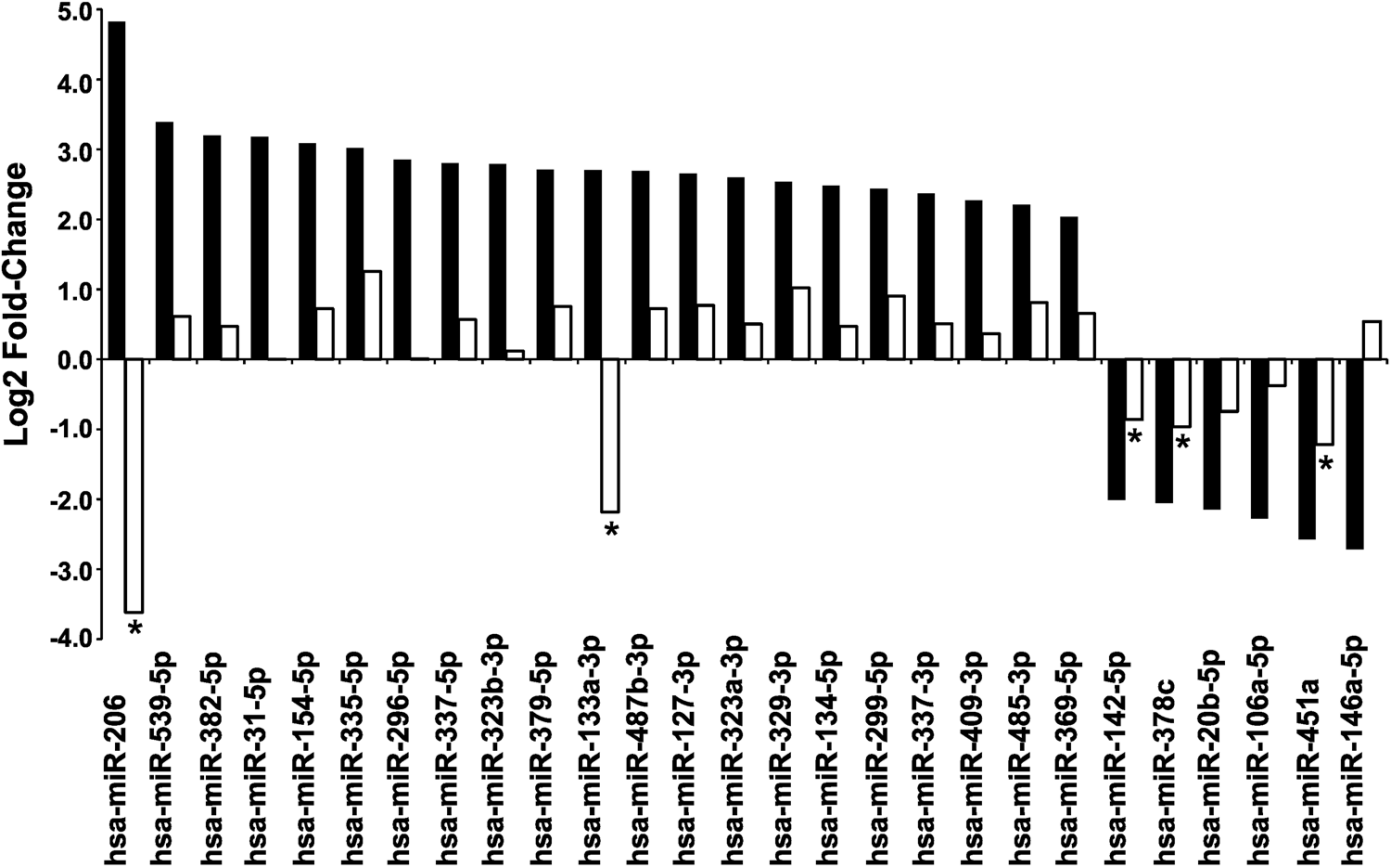
In silico validation of the canonical miRNAs found modulated in our GII + GIII vs GI grade samples. Expression modulation of each miRNA is reported as log2 fold-change value as calculated in GII + GIII vs GI grade cases of our sample collection (black bars) and of a chondrosarcoma public dataset (white bars). *FDR-BH < 0.1.

Among the other 27 miRNAs passing the expression threshold, while seven miRNAs showed no expression difference (|log2FC|<0.5) in GII + GIII vs GI grade samples of the public dataset, 17 miRNAs confirmed the trend of modulation of their expression we found in our samples, even if always with a smaller fold-change value (Fig. 3). On the other hand, three miRNAs showed a modulation trend opposite to that observed in our dataset (hsa-miR-206, hsa-miR-133a-3p, hsa-miR-146a-5p). The highly expressed hsa-miR-451a confirmed its expression reduction in GII + GIII vs GI samples also in the public dataset, and here with a statistically significant FDR value (FDR-BH = 0.02).

### IsomiRs of the differently modulated miRNAs

Most miRNAs can have sequence and length variability, potentially resulting in altered targeting capacity and/or specificity. These isoforms (termed isomiRs) differ from the canonical mature miRNA sequences, as deposited in the miRBase, by the addition or trimming of nucleotides at either end and may also carry internal nucleotide substitutions^13,17,18^.

Globally, by small RNA next-generation sequencing and by applying an ad hoc bioinformatics pipeline, we identified a total of 1576 isomiRs expressed across our nine CCC samples.

Considering the 34 canonical miRNAs with a strong different expression level in our GII + GIII vs GI CCCs reported in Table 2, and setting a threshold of mean expression >0.001% to exclude poorly expressed isomiRs, we identified a total of 57 different isomiRs deriving from 15 miRNAs across the nine CCC cases. They are reported together with their normalized read counts (counts per million, CPM) and sequences in the Supplementary Information (Table S2A), while the isomiR naming rules we implemented here are described in the Supplementary Information (Table S2B).

The expression values for the isomiRs of each miRNA were extremely variable and characteristic of the individual miRNA they derive from. There were miRNAs with many highly expressed different isomiRs, such as the aforementioned miR-451a (with 26 isomiRs), and miRNAs with no isomiRs at all (19 out of the 34 canonical miRNAs had no isomiR passing expression threshold (mean >0.001%)).

Altogether, the cumulative expression of all the 57 isomiRs corresponding to the differently modulated miRNAs represent the 21.9%, 2.4%, and 4.7% of all the isomiRs expressed in GI, GII, and GIII samples, respectively. Among them, the isomiRs of miR-451a were collectively the most abundant in each grade, even if with a different abundance (21.3% in GI, 2.1% in GII, 4.1% in GIII). Moreover, if considering individual isoforms, one isomiR of miR-451a resulted the most abundant isoform over all the isomiRs expressed in GI group (hsa-miR-451a.3.P0.S.57: 14.8% in GI vs 1.6% in GII, 3% in GIII).

Regarding the frequency of the various modifications shown by each individual miRNA, 3′-end variations have a marked preponderance being common to all samples (Table S2A). On the other hand, nine isomiRs deriving from hsa-miR-142-5p, hsa-miR-144-3p, hsa-miR-337-3p, hsa-miR-409-3p, and has-miR-4284 carried the more critical 5′-end shift, mostly represented by the insertion/deletion of one or two C, in some cases coupled with a 3′-end shift.

**Fig. 4:**
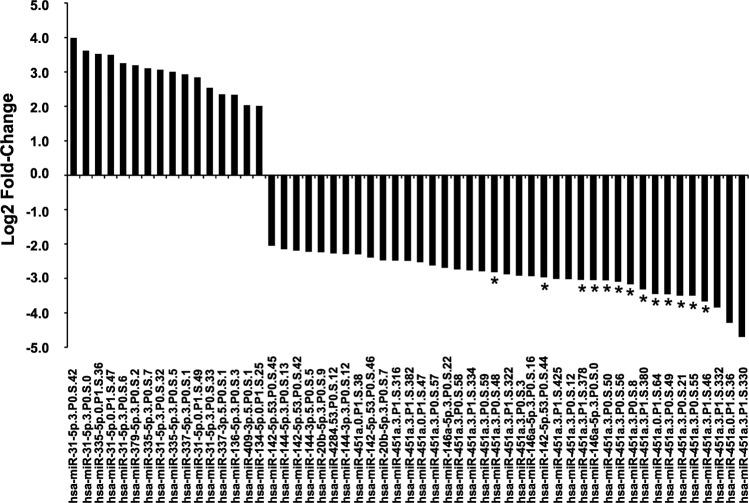
Differently modulated isomiRs in our GII + GIII vs GI CCC cases. Expression modulation of each isomiR is reported as log2 fold-change value. IsomiR names follow nomenclature rules described in Table S2B. *FDR-BH < 0.1.

Next, similarly to what done with canonical miRNAs, we considered among these 57 isomiRs those showing a strongly different expression level in our GII + GIII vs GI CCCs (|log2FC|>2). Globally, we found 55 isomiRs, including 16 more and 39 less expressed in GII + GIII vs GI cases (Fig. 4). All these isomiRs maintained the expression trend (up- or down-) of the miRNA they derive from. Interestingly, two isoforms of hsa-miR-31-5p showed the higher expression increase, while all the 26 isomiRs of hsa-miR-451a showed a reduction in our GII + GIII vs GI grade cases, with 12 isoforms being the most down-expressed (log2FC < −3). Moreover, the reduced expression of 11 of these hsa-miR-451a isomiRs resulted statistically significant (FDR-BH < 0.1).

Then, by using the latest version of MicroRNA Target Prediction Database (miRDB) online database (http://mirdb.org/), we identified the putative target genes specific of these isomiRs. Those genes already recognized by the corresponding canonical miRNAs were excluded from the pathway analysis, thus considering only the nine isomiRs targeting a different set of genes. Interestingly, all these isomiRs were those showing a 5′-end variation. Finally, by using the latest version of Reactome Pathways Database (https://reactome.org/), we defined that the pathways affected only by this specific set of isomiRs in Table 4 are reported the most important pathways differently targeted by this set of isomiRs, many of which are related to oncogenic signaling.

**Table 4:** Selected pathways specifically targeted by the isomiRs with 5′-end variation, with the exclusion of the canonical miRNAs.

IsomiR	Pathway name	Entities found	Entities total	*p*-Value	FDR	Genes
hsa-miR-142-5p.53.P0.S.42; hsa-miR-142-5p.53.P0.S.44; hsa-miR-142-5p.53.P0.S.45; hsa-miR-142-5p.53.P0.S.46; hsa-miR-142-5p.53.P0.S.47	Insulin-like growth factor-2 mRNA binding proteins (IGF2BPs/IMPs/VICKZs) bind RNA	5	13	3.90E−03	6.45E−01	CD44
	GABA receptor activation	12	67	7.59E−03	6.45E−01	ARHGEF9, BTG3, FER, GABRB2, GABRG2, GNAI2, GNG12, KCNJ16, KCNJ3, KCNJ6, NUDT13, RAD51AP1
	Ion channel transport	25	207	2.43E−02	6.45E−01	ANO1, ANO5, ASIC1, ASPH, ATP1B3, ATP6V0A2, ATP6V1C2, ATP8A1, ATP8B2, ATP8B4, BTF3, CALM1, CLCN3, KIAA1324L, MBTPS2, NEO1, PHEX, PLEKHO2, RASAL2, REEP3, SLC4A4, SLC9C2, TRDN, VAPA, WNK3
hsa-miR-144-3p.5.P0.S.5	RUNX3 regulates NOTCH signaling	6	16	1.55E−02	6.46E−01	GALNT1, MAML3, NR6A1, PLEKHG1, RBPJ, RTN4
	Molecules associated with elastic fibers	10	38	2.16E−02	6.46E−01	ARPIN, BMP10, FBN1, FBN2, FN1, ITGA8, ITGB8, MFAP3L, PCDH18, RICTOR
hsa-miR-337-3p.5.P0.S.1	Signaling by receptor tyrosine kinases	36	622	4.78E−03	2.53E−01	CBL, CDC73, ERBB4, ESYT2, FGFR2, FGFR3, HNRNPH1, JAK2, KRAS, MEF2C, PDGFD, PGM2L1, PIK3CB, PRKAA2, PRKCB, PTPRK, RALB, RNF41, RPS6KA3, SPRED1, STAM, STAT3, USP8, ZFX
hsa-miR-409-3p.5.P0.S.1	Cilium assembly	24	208	6.75E−03	6.27E−01	ATP6V0A2, BBIP1, C5, CCDC88A, CDC27, CEP41, CNGB1, CSNK1A1, DYNC2H1, DYNC2LI1, DYNLRB2, ERCC4, GADL1, GPATCH2L, ITIH5, KLHL32, LIN54, LZTFL1, MKKS, NAT10, RPGRIP1L, SEMA6D, SEPT2, TUBB1
	Striated muscle contraction	7	40	1.80E−02	6.27E−01	ACTN2, ENPP2, PHAX, PRRC2B, STUM, TMOD2, TMOD3
	Frs2-mediated activation	4	17	2.70E−02	6.27E−01	FRS2, GM2A, MAPK1, YWHAB
hsa-miR-4284.53.P0.S.12	Signaling by TGF-beta family members	13	114	7.48E−04	3.04E−01	ACVR1B, CDK8, FNDC3A, GRIP2, HDAC1, INHBA, NEDD4L, PMEPA1, SMAD2, SMAD5, STUB1, TFDP2, XPO1
	Chromatin organization	20	256	3.49E−03	4.15E−01	AFF4 Q9H9L4 EIF1B Q12962 EP400 Q96L91 GATAD2B, HDAC1, JADE2, KDM4C, MSL3, MTA3, NCOA2, PRMT3, PTPRN, REST, SETD2, SSR3, SUPT7L, TBL1XR1, WDR5B, ZZZ3
	GABA receptor activation	7	67	1.82E−02	5.08E−01	ADCY3, GABRA5, GABRB2, GNG13, NPTN, POC1B, STAM
	Activation of RAC1	3	15	2.22E−02	5.08E−01	PAK5, SOS1

## Discussion

In the current study, a systematic also if somewhat limited small RNA profiling was performed to identify the miRNAs and isomiRs expressed in a small cohort of nine histologically validated and reliably graded conventional central chondrosarcoma tumors. The miRNA profiles of CCC samples were produced by small RNA next-generation sequencing technology and analyzed by focusing our attention on miRNAs distinguishing advanced GII and GIII cases from GI patients, in order to highlight any possible tumor progression-linked alteration. This study concerns only the microRNAs, neglecting all the other species of small and long non-coding RNAs that could participate, both directly and indirectly, in regulating gene expression.

We identified 290 miRNAs expressed across all the CCC samples analyzed. The analysis of the 20 most abundantly expressed miRNAs in Grade I, Grade II, and Grade III cases showed that they represent about 80% of the total miRNA counts.

The most abundant miRNA in all histological grades of CCC was hsa-miR-140-3p, thus resulting not significantly altered by tumor progression. It is known that this miRNA is positively regulated by the transcription factor Sox9 during cartilage differentiation^19,20^. Other two miRNAs highly expressed in all grades were hsa-miR-23b-3p, known for contributing to chondrocyte differentiation^21^, and hsa-miR-21-5p, which is one of the commonly overexpressed miRNAs in cancer and is an oncomiR regulating cell proliferation, migration, invasion, and drug resistance^22^. We also found two miRNAs most abundantly expressed in GI tumors, which are hsa-miR-451a and hsa-miR-16-5p. These miRNAs possess oncosuppressor properties: hsa-miR-16-5p targets SMAD3 in chondrocytes^23,24^, while hsa-miR-451 has tumor suppressor activity in many cancers^25^ and decreases cell chemoresistance targeting MDR1 gene^26^.

Considering the other miRNAs, hsa-miR-99a-5p is associated with poor prognosis in acute myeloid leukemia (AML) and its ectopic expression upregulates genes that are downstream targets of E2F and MYC and that are critical for stem cells maintenance and cell cycle^27^. Hsa-miR-103a-5p targets the known tumor suppressor transcription factor KLF4 in many cancers, including colorectal cancer^28^; moreover, it belongs to a group of miRNAs with mechanosensitive properties and is involved in bone formation and differentiation^29^. The let-7f-5p miRNA has been found to inhibit apoptosis and to promote chemotherapeutic resistance in some tumors^30,31^.

The most less expressed miRNAs we found in our GII + GIII tumors as compared with GI cases (Log2FC < −2.5) included hsa-miR-451a, hsa-miR-146a-5p, and hsa-miR-489-3p (even if not reaching statistical significance probably due to the limited number of cases). According to our data these less expressed miRNAs have tumor suppressor function in many tumor types^25,32,33^ and mainly participate to biological pathways involved in apoptosis, in the downregulation of ERBB2 signaling, of MAP kinase activation, transmembrane receptors that regulate cell migration, the degradation of GLI2/3 transcription factors, insulin receptor signaling and WNT signaling. These pathways, all endowed with established oncogenic potential, would be enhanced in advanced tumors where the expression of their regulating miRNAs was strongly reduced, thus supporting the establishment of the tumor malignant phenotype. In particular, the canonical WNT signaling pathways has been implicated in the β-catenin-dependent regulation of mitotic and cell-fate-determining gene transcription of osteoarthritis^34^. The Dickkopf WNT signaling pathway inhibitor 1 (DDK1) gene, an antagonist of canonical WNT/β-catenin signaling, and β-catenin were progressively overexpressed in chondrosarcoma tissues with increasing histological grade and correlated with poor prognosis^35^. Targeting of WNT signaling has been proposed as being of interest for chondrosarcoma therapeutic treatment.

On the other hand, when analyzing the miRNAs with a higher expression in GII + GIII cases as compared with GI tumors, the most increased miRNAs (Log2FC > 3) included hsa-miR-206, hsa-miR-31-3p, hsa-miR-539-5p, hsa-miR-382-5p, hsa-miR-31-5p, hsa-miR-154-5p, and hsa-miR-335-5p. The most interesting pathways targeted by these miRNAs were (i) apoptotic execution phase; (ii) syndecan interactions; (iii) interferon signaling; (iv) regulation of RUNX1 expression and activity. Interferons are a family of cytokines able to trigger a potent anti-viral response in the cells^36^. Syndecans are a small family of heparan sulfate pro-teoglycans, which are involved in different pathologies including cancer. Syndecans have been recently involved in tumor progression. For example, syndecan-1 acts as a tumor suppressor in breast cancer cells^37,38^. As well, some authors found that syndecan-4 inhibited breast carcinoma cell invasion and was associated with good prognosis^39,40^. On the other hands, contrasting findings are reported since the expression of syndecan-4 has been found correlated with negative estrogen receptor status^41^. RUNX proteins are transcription factors crucial for development and normal tissue homeostasis and have been reported as both oncogene and tumor suppressors in different tumors^42^. RUNX1 expression decrease is also correlated with the loss of its oncosuppressor function and the induction of epithelial-mesenchymal transition^43^.

Interestingly, hsa-miR-451a, which is the less expressed miRNAs in the high-grade group of our small set of chondrosarcoma, was also significantly validated with the same trend of modulation in the public dataset. Hsa-miR-451a, when expressed at low level in chondrosarcoma could represent a potential biomarker capable of detecting GI cases from the more advanced GII and GIII and help to choice the surgical strategy often critical in the management of these patients.

Each individual miRNA exists as a set of naturally occurring sequence variants, called isomiRs, differing from the canonical sequence by 3′- and/or 5′-end length variations and also internal nucleotide modifications. These subtle sequence variations can have profound effects on miRNA function. With regard to the frequencies of the various isomiR modification types shown in this study, marked preponderance of 3′-end variations is common to all tumor samples of our collection, while the more critical 5′-end shifts are less frequent. The nucleotide modifications, either alone or in combination with end shifts, are quite frequent, accounting for almost one-third of total isomiRs. Although many isomiRs may affect the same downstream targets as their canonical sequences^44^, 3′-end modifications mainly modulate miRNA processing, stability and targeting effectiveness^45–48^, while 5′-end modifications altering the seed sequence affect miRNA targeting specificity. Analyzing the behavior of this latter group of isomiRs we observed that the expression of six of them was lower, while in two cases was higher in GII + GIII vs GI tumors. Studying the genes targeted by the set of less expressed isomiRs we found that many of the pathways targeted by these isomiRs are correlated with oncogenic signaling. Our findings suggest that this sets of isomiRs collaborate with the canonical miRNAs to repress these tumor-linked pathways.

One of the targets of the hsa-miR-142-5p isoforms is CD44, which is a tumor stem cell marker and receptor for hyaluronan, collagens, and matrix metalloproteinases in various tissues acting also as a cofactor for VEGF and FGF2 binding. Concordantly, CD44 expression is reported as increasedin chondrosarcomas and correlates with the increasing grading and metastatic potential^49^. It is to note that the hsa-miR-142-5p is also significantly modulated with the same decreasing trend in the public dataset.

The isomiR-4284.53.P0.S.12, which is less expressed in our dataset, targets TGF-β family members, known to be involved in a wide range of cellular processes, such as proliferation, differentiation, migration, and death. They are key regulators of normal chondrogenesis, and this signaling pathways may be involved in the development and progression of central chondrosarcoma. In addition, other authors demonstrated that TGF-β signaling is higher in high-grade chondrosarcoma than in low-grade chondrosarcoma^50,51^. In the same way, isomiR-4284.53.P0.S.12 and the hsa-miR-142-5p isoforms, all downregulated in our dataset, target the activation of Gamma-aminobutyric acid (GABA) receptor. GABA, being a neurotrophic factor shows a crucial function in the development of neural crest, thus playing important functional roles in the proliferation of chondrosarcoma cells, which are derived from neural crest cells^52^. The less expressed isomiR-4284.53.P0.S.12 targets also the activation of Rac1 pathway. Rac1 is a Rho GTPases regulating many intracellular signaling pathways, including those involved in tumorigenesis, invasion, and metastasis. It has been also demonstrated that the overexpression of Rac1 resulted in an accelerated tumorigenic process in colorectal cancer as well as in other solid tumors^53,54^.

There are several publications in the literature that describe the behavior of miRNAs in CCC. To facilitate the comparison with our data we have listed them in the Supplementary Information (Table S3), reporting only the publications dealing with tumor tissues, with an evaluation of their agreement with our findings.

In conclusion, this study, although involving a small number of patients, suggests that the contribution of miRNA deregulation to the progression of CCC could be important and strongly indicates that when studying miRNA deregulation in tumors, not only the canonical miRNAs, but the whole set of corresponding isomiRs should be taken in account.

## Materials and methods

### Study cohort and patient characteristics

Specimens used for small RNA next-generation sequencing consisted of fresh-frozen tumor tissues from nine patients (listed in Table 1), diagnosed with primary CCC from the Department of Surgical Pathology at the ASST Orthopaedic Traumatology Centre Gaetano Pini-CTO, Milan, Italy. The enrolled patients were re-evaluated by two sarcoma pathologists (A.P. and E.A.) and the lesions were histologically classified in accordance with the World Health Organization (WHO) classification of soft tissue and bone tumors 2002/2013^55^.

Patients were included in this study based on a histological diagnosis of Grade I—low grade, low cellularity, mostly chondroid matrix, with uniform hyperchromatic plump nuclei with occasional binucleations^56^; Grade II—high grade, increased cellularity, nuclear atypia, hyperchromasia, and nuclear size; Grade III—high grade, high cellularity, nuclear pleomorphism with mitoses present (Fig. 1).

The study was approved by the institutional Internal Review Board (Department of Surgical Pathology of the ASST (Local Health and Social Care Company) Orthopaedic Traumatology Centre Gaetano Pini-CTO, Milan, Italy, Protocol, ASST# HUM00026026). Informed consent was obtained from all patients prior to biopsy. Eligibility criteria for the study include patients before surgery and from surgical resection. Clinical features, imaging and histological data were used without any information linked to patients’ identities. Local radiographs were performed at each evaluation.

Open biopsies were snap frozen in optimal cutting temperature (OCT), and longitudinal sections were cut. Hematoxylin and eosin-stained frozen sections were reviewed by the study pathologists (A.P. and E.A.) to identify cores with the highest tumor content that were subsequently used for nucleic acid extraction.

### RNA isolation

Total RNA was isolated from human chondrosarcoma tissue samples embedded in OCT compound using Trizol reagent (Thermofisher), according to the manufacturer’s protocol, and the RNA concentration was measured by NanoDrop1000 Spectrophotometer (ThermoFisher Scientific, Waltham, MA, USA). The RNA quality was checked by 2100 Bioanalyzer (Agilent Technologies, Santa Clara, CA, USA) using an Agilent RNA 6000 Nano kit and shown in the Supplementary Information (Fig. S2).

### Small RNA library construction and next-generation sequencing

Total RNA samples (about 3 µg each) were enriched for small RNAs up to 200 bp by size selection using the FlashPage small RNA Isolation Kit (Ambion, Thermofisher) and miRNA quality and enrichment was assessed by the 2100 Bioanalyzer (Agilent Technologies) using a Agilent Small RNA kit. Enriched RNA samples were processed using the Small RNA Expression Kit (Applied Biosystems, ThermoFisher Scientific), according to the manufacturer’s protocol. Briefly, RNA was first hybridized and ligated with the adapter mix “A”, subsequently reverse transcribed and treated with RNAse H. The cDNA libraries were then PCR amplified, purified and size-selected by PAGE, resulting in libraries containing inserted small RNA sequences of 20–40 bp. Size, integrity and purity of the libraries were verified by the 2100 Bioanalyzer (Agilent Technologies), using the DNA 1000 kit. The cDNA libraries were barcoded using the SOLiD RNA barcoding kit and amplified onto beads using emulsion PCR. Templated beads were deposited on slides and sequenced using the Applied Biosystems SOLiD 5500xl Sequencer, obtaining about 40 millions of 50-bp reads per sample.

### Quantification of known microRNAs

The qualified clean reads were mapped and analyzed with the ‘small RNA’ bioinformatics pipeline from the Lifetech Lifescope version 2.5.1 software, using as a target the human genome GRCh38/hg38 and the dataset of mature and precursor miRNA sequences (miRBase, release 21)^57^. Any sequence match against repetitive elements of the genome (SINE, LINE, etc.), and against non-miRNA small RNAs (snoRNAs, piRNAs, tRNAs, rRNA fragments, etc.) were filtered out from the results.

Sequence counts were extracted and reformatted with Perl scripts from the pipeline output. Differential expression analysis of our high- vs low-grade CCC cases was performed by comparing GII + GIII cases to GI cases using the EdgeR Bioconductor package (v.3.28.1) in R environment (v.3.6.0)^58^. miRNAs with mean normalized count per million (CPM) < 10 across all the nine samples were discarded to exclude poorly expressed genes. After having estimated the tagwise dispersion, genewise exact test^59^ as implemented in EdgeR was used to measure the statistical significance of differential expression. MiRNAs were defined as significantly differentially expressed if their false discovery rate (*p*-value corrected for multiple comparison with the Benjamini–Hochberg procedure (FDR-BH)) was <0.1.

### Identification and differential expression of isomiRs

The alignment files in BAM format corresponding to the same biological group were merged and converted to SAM (Sequence Alignment/Map) format with samtools^60^. The files were then processed and analyzed with the miRDeep2 software for miRNA prediction^61^. The differential expression analysis for isomiRs was carried out by EdgeR Bioconductor package, with the same analytical strategy described previously (significant FDR-BH < 0.1).

### In silico validation in a chondrosarcoma public dataset

Canonical miRNAs identified in our CCC cases were validated in a recently published dataset including 73 human chondrosarcoma samples of different grades sequenced for miRNA profile by Illumina technology (E-MTAB-7265)^3^. After downloading and quality control of fastq files, reads were adapter trimmed, filtered, and mapped on the human reference genome GRCh38/hg38 by using miRDeep2 tool (v.0.1.3). The same tool was used to generate raw counts for mature known miRNAs annotated in miRBase 21. Raw counts were imported in EdgeR Bioconductor package (v.3.28.1) and used to perform a differential expression analysis of GII + GIII (*n* = 56) vs GI (*n* = 17) cases, similarly to what done for our cases. miRNAs with mean CPM < 10 across the whole public dataset were discarded before testing for statistical significance. A FDR-BH < 0.1 was set as threshold to define statistically significant differentially expressed miRNAs in high- vs low-grade cases of the public dataset.

## Supplementary information

Figure S1

supplementary figure legend

Table S1

Table S2A

Table S2B

Table S3

## Data Availability

Sequencing data have been submitted to the European Nucleotide Archive on September 7, 2018, with the Accession number PRJEB28552.
