# Supplementary figures and images for: Expression profiling of microRNAs and isomiRs in conventional central chondrosarcoma
Source: Cell Death Discov. 2020 Jun 10;6:46. doi: 10.1038/s41420-020-0282-3 (PMC7287106; doi:10.1038/s41420-020-0282-3)

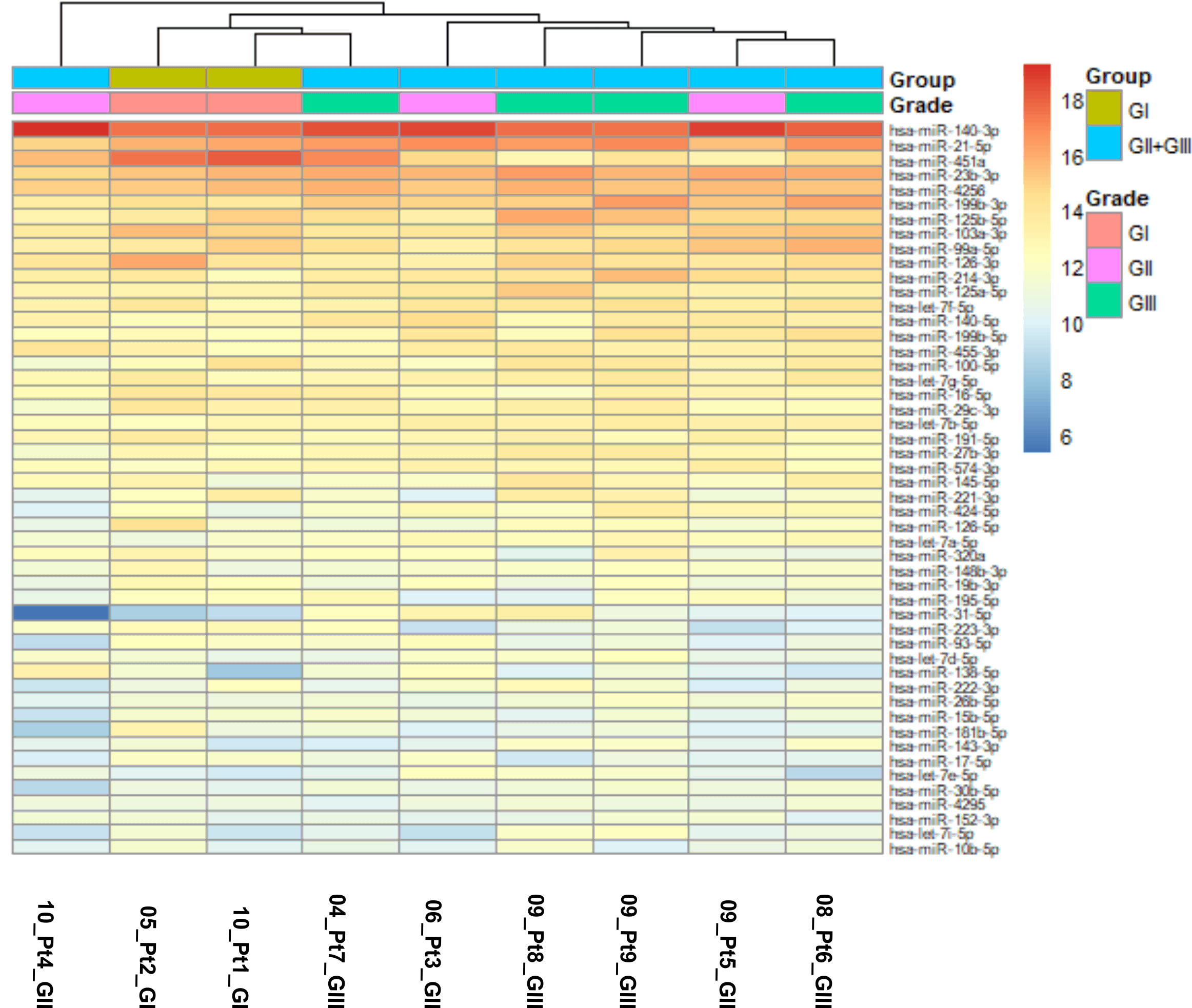

Supplement: Supplementary file 1 — Figure S1 [file 41420_2020_282_MOESM1_ESM.tif]
