# Supplementary material for: Expression profiling of microRNAs and isomiRs in conventional central chondrosarcoma
Source: Cell Death Discov. 2020 Jun 10;6:46. doi: 10.1038/s41420-020-0282-3 (PMC7287106; doi:10.1038/s41420-020-0282-3)
Supplement: Supplementary file 2 — supplementary figure legend [file 41420_2020_282_MOESM2_ESM.docx]

Fig. S1. **Clustering of the nine CCC samples according to the expression of the first 50 most expressed miRNAs.** Heatmap shows the normalized read counts (log2 CPM) of each miRNA across all samples as a colour gradient ranging from blue to red.
