# Supplementary material for: Expression profiling of microRNAs and isomiRs in conventional central chondrosarcoma
Source: Cell Death Discov. 2020 Jun 10;6:46. doi: 10.1038/s41420-020-0282-3 (PMC7287106; doi:10.1038/s41420-020-0282-3)
Supplement: Supplementary file 3 — Table S1 [file 41420_2020_282_MOESM3_ESM.docx]

| **Supplementary Table S1. List of miRNAs having an expression level above 0.001% of the total normalized counts (CPM) in at least five samples.** | | | | | | | | |  |
| --- | --- | --- | --- | --- | --- | --- | --- | --- | --- |
|  | **GI** | | **GII** | | | **GIII** | | | |
|  | **Pt1** | **Pt2** | **Pt3** | **Pt4** | **Pt5** | **Pt6** | **Pt7** | **Pt8** | **Pt9** |
| *hsa-miR-140-3p* | 193262.69817 | 173650.90558 | 408243.76949 | 603639.53187 | 414300.87073 | 241685.11125 | 312457.92889 | 208664.81084 | 173293.99906 |
| *hsa-miR-451a* | 263272.14584 | 173326.60819 | 27736.03256 | 46369.80703 | 8017.08854 | 27533.19985 | 121744.07471 | 7284.21919 | 18497.84742 |
| *hsa-miR-21-5p* | 67642.41812 | 55376.19533 | 107492.58658 | 28717.53910 | 44443.01025 | 106590.61636 | 87295.12950 | 85020.42169 | 119520.43186 |
| *hsa-miR-23b-3p* | 53789.95534 | 39567.73227 | 52453.63044 | 27730.25318 | 66905.61091 | 63426.30838 | 60773.14814 | 83288.77467 | 51037.12307 |
| *hsa-miR-4256* | 46742.57924 | 35393.95579 | 34687.28309 | 32915.86645 | 47539.08673 | 38564.65133 | 58165.97260 | 58777.08824 | 39690.48857 |
| *hsa-miR-199b-3p* | 15298.87623 | 21253.89933 | 30405.81424 | 12127.54293 | 40153.84150 | 77217.10009 | 36847.19674 | 32687.93877 | 86405.15851 |
| *hsa-miR-103a-3p* | 29709.32802 | 46092.31988 | 16193.07335 | 13965.54717 | 34266.72977 | 42028.11217 | 15162.12119 | 34841.05313 | 20422.86726 |
| *hsa-miR-125b-5p* | 30933.26920 | 13292.74326 | 9979.87067 | 8195.26419 | 26668.28501 | 27469.25125 | 22865.78323 | 71016.30000 | 43785.05638 |
| *hsa-miR-126-3p* | 16959.75138 | 67140.60086 | 9035.84189 | 15710.82169 | 14742.08983 | 23364.03552 | 10007.73337 | 27969.16221 | 18326.43501 |
| *hsa-miR-99a-5p* | 32471.20624 | 13118.86466 | 9359.52351 | 10485.76533 | 40670.36477 | 57279.11652 | 20546.38673 | 17827.35219 | 26823.24616 |
| *hsa-miR-214-3p* | 5943.95193 | 15149.51835 | 14446.38157 | 10778.12765 | 23873.99183 | 17448.89480 | 12700.78565 | 17809.51317 | 45652.30309 |
| *hsa-let-7f-5p* | 6127.20834 | 16165.88019 | 15846.38927 | 8854.53519 | 11016.48954 | 17382.09700 | 9458.39528 | 16995.01114 | 19219.25311 |
| *hsa-miR-125a-5p* | 8350.97449 | 8758.78967 | 15217.21976 | 8482.62746 | 9426.83304 | 10134.85189 | 13307.43991 | 35113.08441 | 13842.14777 |
| *hsa-miR-140-5p* | 7853.21410 | 5714.53411 | 23210.26427 | 9120.52887 | 15447.34276 | 10655.77979 | 16616.56447 | 6155.36625 | 13480.51310 |
| *hsa-miR-199b-5p* | 5022.64835 | 6374.85881 | 16985.43730 | 5165.39731 | 14684.91673 | 21423.41687 | 6236.34561 | 5673.46581 | 19814.79745 |
| *hsa-miR-16-5p* | 15243.72893 | 15860.21264 | 6355.23108 | 6145.76146 | 8373.53068 | 6996.83249 | 12840.91353 | 3893.24178 | 11253.49537 |
| *hsa-miR-100-5p* | 20104.10375 | 5458.54617 | 4217.62991 | 2949.38759 | 8022.32956 | 14075.81677 | 7450.18355 | 17145.79199 | 14645.59737 |
| *hsa-miR-455-3p* | 5089.78159 | 9661.99241 | 11796.51644 | 18084.43899 | 9522.72945 | 10788.00356 | 6480.82405 | 14669.59933 | 9500.06131 |
| *hsa-let-7g-5p* | 5969.68734 | 13735.02971 | 9575.95494 | 6926.76769 | 8369.79469 | 14318.57877 | 7425.85831 | 11308.04257 | 15206.33915 |
| *hsa-miR-29c-3p* | 8831.30893 | 15947.84194 | 7034.77882 | 3126.49697 | 5810.49729 | 5888.54823 | 10226.40977 | 11200.98103 | 12135.43496 |
| *hsa-miR-191-5p* | 7929.61328 | 13304.47317 | 7756.63974 | 7699.36891 | 11178.73083 | 6319.40990 | 6313.72384 | 10002.88526 | 6090.23296 |
| *hsa-let-7b-5p* | 7743.33797 | 4527.05365 | 11358.90162 | 5461.44026 | 10267.94512 | 10263.96265 | 7389.74661 | 9136.32074 | 12539.63018 |
| *hsa-miR-27b-3p* | 5345.52157 | 7876.97675 | 8440.56080 | 3125.23347 | 7532.73731 | 5284.41328 | 7957.78141 | 14102.81262 | 13293.45470 |
| *hsa-miR-574-3p* | 6891.79796 | 4195.85631 | 9089.59004 | 5498.30142 | 11947.05300 | 4656.85161 | 7606.33321 | 7839.36937 | 6871.40546 |
| *hsa-miR-145-5p* | 2484.19888 | 8319.95319 | 4016.38200 | 6049.68072 | 4136.31172 | 11175.70519 | 4053.00885 | 16630.82076 | 7574.04463 |
| *hsa-miR-126-5p* | 3436.82732 | 19337.09472 | 2319.86021 | 1668.36598 | 3034.93721 | 4173.27983 | 2331.35014 | 5760.52021 | 5946.21187 |
| *hsa-miR-221-3p* | 12544.47054 | 4596.74309 | 1031.43814 | 1396.82390 | 2379.79263 | 3629.24180 | 3531.03319 | 12779.76140 | 8936.19900 |
| *hsa-miR-424-5p* | 1609.04566 | 5167.36851 | 6669.10587 | 1056.28387 | 7305.56764 | 6952.35338 | 3866.23669 | 4198.67318 | 12545.22113 |
| *hsa-miR-320a* | 4465.55604 | 8435.18227 | 4646.25199 | 5605.25923 | 2233.48686 | 1728.61741 | 5171.91426 | 1296.89648 | 9063.66395 |
| *hsa-let-7a-5p* | 3597.72591 | 2170.72257 | 6983.51077 | 2994.48895 | 5948.55200 | 6648.12263 | 4342.57119 | 5737.43927 | 7679.70922 |
| *hsa-miR-19b-3p* | 4969.98194 | 6999.99379 | 5124.91153 | 1771.09382 | 2457.54025 | 3529.99483 | 2317.39030 | 2176.66186 | 4643.91099 |
| *hsa-miR-148b-3p* | 1992.89476 | 7878.35674 | 3536.70012 | 2768.65252 | 3802.17931 | 3608.66428 | 2804.03446 | 4005.65502 | 4532.69879 |
| *hsa-miR-195-5p* | 5261.11184 | 4738.88195 | 1008.98471 | 1619.91360 | 5459.77424 | 2766.99085 | 7017.51189 | 1289.70598 | 4813.37306 |
| *hsa-miR-223-3p* | 6624.19159 | 6017.44168 | 642.53555 | 3815.98203 | 549.19109 | 995.89932 | 4959.81375 | 1749.98004 | 2423.39437 |
| *hsa-miR-93-5p* | 4304.65746 | 5434.39636 | 5921.17549 | 511.99114 | 1067.37874 | 1989.63537 | 3403.38836 | 1676.75774 | 2506.91186 |
| *hsa-miR-138-5p* | 278.57604 | 2942.13637 | 4897.61309 | 9526.77076 | 1175.49238 | 741.05462 | 2914.01353 | 1136.64722 | 2766.13250 |
| *hsa-let-7d-5p* | 1718.59300 | 3058.05544 | 3915.68231 | 3384.08565 | 1717.61872 | 2116.00246 | 1890.45861 | 3788.54047 | 5216.13804 |
| *hsa-miR-222-3p* | 4803.22488 | 2014.09383 | 3467.40874 | 668.06054 | 962.80632 | 2132.57000 | 1460.40617 | 6218.76337 | 3239.28275 |
| *hsa-miR-31-5p* | 521.88173 | 365.69706 | 8175.60647 | 40.59672 | 1272.71675 | 1032.51676 | 4556.17635 | 11038.64598 | 2075.32533 |
| *hsa-miR-181b-5p* | 1942.20109 | 8314.43324 | 1032.42261 | 337.79330 | 1094.23894 | 1303.66519 | 2580.50972 | 1682.98767 | 2478.48038 |
| *hsa-miR-15b-5p* | 3368.85716 | 3359.58302 | 2734.69974 | 650.53638 | 1311.70565 | 2344.41296 | 3383.88358 | 1233.55425 | 2929.87360 |
| *hsa-miR-17-5p* | 3428.06951 | 4195.16631 | 3855.00503 | 963.60908 | 1196.36791 | 1336.37817 | 2130.67386 | 758.21308 | 1970.70092 |
| *hsa-miR-26b-5p* | 2002.84817 | 2631.63887 | 1592.28180 | 1176.59080 | 2614.27493 | 3372.97250 | 2658.44524 | 2600.37966 | 4238.41554 |
| *hsa-miR-143-3p* | 738.91399 | 2728.23809 | 1331.53039 | 1390.50641 | 1351.38509 | 4172.01352 | 888.49827 | 4258.61227 | 3743.03152 |
| *hsa-miR-4295* | 2005.41872 | 1954.06431 | 2156.23032 | 2232.81973 | 1874.38881 | 2905.33511 | 1262.34910 | 2532.92074 | 1931.99769 |
| *hsa-miR-152-3p* | 1348.31403 | 2549.52953 | 1355.04403 | 2619.94436 | 3051.88198 | 1160.99437 | 1749.88491 | 1683.20723 | 3051.05416 |
| *hsa-let-7e-5p* | 806.07712 | 1256.47991 | 5140.05719 | 1997.04012 | 1492.21966 | 468.42883 | 1383.52949 | 3743.22937 | 3499.45688 |
| *hsa-miR-30b-5p* | 1386.99186 | 2070.67337 | 1846.27446 | 481.55733 | 2164.75166 | 2886.12943 | 2580.70477 | 2335.04488 | 3192.77819 |
| *hsa-miR-10b-5p* | 1178.50818 | 3278.85367 | 1268.50553 | 1196.58702 | 1834.07194 | 2399.60293 | 1634.02654 | 3655.98286 | 1028.69114 |
| *hsa-let-7i-5p* | 714.88232 | 2826.90730 | 551.47229 | 619.55323 | 1334.47573 | 2177.04911 | 1352.37757 | 3913.02936 | 4927.53243 |
| *hsa-miR-218-5p* | 756.60895 | 4765.10174 | 553.10044 | 1257.23490 | 586.72809 | 2133.36145 | 633.06930 | 2727.44835 | 1957.13528 |
| *hsa-let-7c-5p* | 2068.42712 | 318.08745 | 1323.90077 | 829.12900 | 2484.66605 | 4280.70505 | 1254.10137 | 1312.15569 | 2719.45458 |
| *hsa-miR-484* | 1608.77665 | 2835.87723 | 700.46768 | 1200.26764 | 932.17471 | 1653.11373 | 1517.66662 | 1972.28163 | 1764.78933 |
| *hsa-miR-101-3p* | 2406.99267 | 1512.46785 | 1197.03696 | 626.03552 | 1815.58674 | 999.38167 | 1423.96010 | 1155.11746 | 2162.00670 |
| *hsa-miR-425-5p* | 1470.29566 | 2741.34799 | 1661.78144 | 1582.55802 | 1201.57351 | 892.43132 | 887.88527 | 1193.70463 | 960.47291 |
| *hsa-miR-142-5p* | 3670.26926 | 2461.90023 | 374.77927 | 1169.77890 | 255.48177 | 1074.83259 | 1581.92092 | 477.37207 | 406.10221 |
| *hsa-miR-10a-5p* | 1169.00312 | 2210.05225 | 926.45981 | 1193.62055 | 818.16493 | 1436.73317 | 530.83641 | 2542.55381 | 2460.36397 |
| *hsa-miR-25-3p* | 1580.17179 | 2619.90896 | 1532.26714 | 984.53919 | 543.02935 | 970.15104 | 1309.60639 | 1458.40818 | 1135.04918 |
| *hsa-miR-34a-5p* | 2143.66059 | 1077.77135 | 620.91512 | 1635.51505 | 1160.35363 | 1647.36258 | 835.55674 | 2248.34726 | 1525.41873 |
| *hsa-miR-30d-5p* | 830.04901 | 2323.90134 | 928.92098 | 471.55922 | 1639.10973 | 1310.47160 | 1179.09157 | 1564.89339 | 2465.13144 |
| *hsa-miR-532-5p* | 1030.70147 | 1866.43501 | 1026.78085 | 773.42524 | 966.82561 | 1746.24021 | 499.93527 | 2103.08278 | 654.44433 |
| *hsa-miR-30e-5p* | 987.98847 | 1486.93806 | 835.98344 | 435.57701 | 1712.60788 | 1310.52436 | 883.12053 | 829.92592 | 1568.93277 |
| *hsa-miR-185-5p* | 1662.13054 | 1676.68654 | 242.59555 | 2022.96928 | 303.90804 | 260.49033 | 1035.39710 | 804.48474 | 555.75760 |
| *hsa-miR-15a-5p* | 1215.72140 | 1484.17808 | 628.48795 | 294.77945 | 1026.95564 | 1022.86115 | 1277.78574 | 435.90322 | 1347.63524 |
| *hsa-miR-497-5p* | 1188.67083 | 1371.70899 | 420.36770 | 305.71145 | 1099.58619 | 1216.55368 | 1530.12181 | 333.56216 | 1208.25160 |
| *hsa-miR-22-3p* | 1008.13442 | 1573.87736 | 723.77306 | 466.56017 | 435.62396 | 575.53747 | 1225.15071 | 932.45910 | 1383.82471 |
| *hsa-miR-331-3p* | 687.17417 | 1658.74668 | 453.65028 | 707.61351 | 835.05658 | 807.79966 | 539.92006 | 1255.64718 | 1244.00766 |
| *hsa-miR-192-5p* | 894.01389 | 1575.94735 | 534.81206 | 984.09971 | 610.33036 | 360.95085 | 565.47132 | 2054.03921 | 187.57848 |
| *hsa-miR-193b-3p* | 400.88647 | 1684.27648 | 848.04317 | 647.40511 | 547.10176 | 661.80478 | 691.10994 | 1101.05152 | 1539.89451 |
| *hsa-miR-130b-3p* | 794.86832 | 1176.44055 | 947.72053 | 842.75280 | 688.75082 | 1136.24858 | 475.21993 | 1101.35341 | 831.79441 |
| *hsa-miR-378c* | 617.94862 | 2967.66617 | 239.49069 | 325.98234 | 356.63690 | 633.62940 | 328.71120 | 677.96495 | 461.27490 |
| *hsa-miR-210-3p* | 973.40209 | 316.01746 | 441.30657 | 1650.67701 | 302.66862 | 506.78744 | 1954.85224 | 473.42004 | 1510.11946 |
| *hsa-miR-301a-3p* | 373.41744 | 991.52204 | 2036.04954 | 594.61289 | 662.61657 | 757.78045 | 196.94251 | 787.11228 | 1075.19570 |
| *hsa-miR-30a-5p* | 548.30461 | 1909.90466 | 453.04445 | 326.36688 | 298.22438 | 832.49269 | 571.54566 | 1052.69407 | 820.26579 |
| *hsa-miR-19a-3p* | 920.97479 | 1395.85879 | 1108.81352 | 346.80258 | 446.54864 | 364.11662 | 469.64714 | 359.14056 | 981.62316 |
| *hsa-miR-144-3p* | 1571.41399 | 1368.25901 | 335.57095 | 149.47724 | 14.69609 | 149.42447 | 1217.51598 | 95.78179 | 224.76479 |
| *hsa-miR-29a-3p* | 487.38852 | 1226.12015 | 355.73361 | 244.45929 | 301.85413 | 354.09167 | 1219.04850 | 757.71907 | 710.26714 |
| *hsa-miR-186-5p* | 593.55827 | 773.48379 | 557.53055 | 302.90978 | 689.37053 | 817.82461 | 460.86999 | 493.48208 | 1060.41652 |
| *hsa-miR-181a-3p* | 546.83999 | 1440.01844 | 130.10119 | 161.34313 | 307.66174 | 535.33216 | 952.16743 | 258.83040 | 648.28996 |
| *hsa-miR-483-5p* | 7.44264 | 809.36350 | 1297.94490 | 1047.60419 | 200.98230 | 392.13371 | 14.18276 | 793.39710 | 879.46917 |
| *hsa-miR-98-5p* | 225.61073 | 816.95344 | 1002.98324 | 498.80682 | 51.66649 | 647.50604 | 253.61782 | 1037.32507 | 792.09435 |
| *hsa-miR-107* | 514.82766 | 1326.16935 | 239.90720 | 150.90554 | 551.10335 | 606.98415 | 185.76906 | 606.25210 | 327.30885 |
| *hsa-miR-374b-5p* | 391.88954 | 779.69374 | 604.51995 | 357.13030 | 387.17998 | 534.96282 | 364.04271 | 608.63979 | 947.99079 |
| *hsa-miR-106b-5p* | 606.50070 | 843.86322 | 1126.40141 | 172.76954 | 230.78172 | 297.42434 | 506.98486 | 277.82209 | 492.30683 |
| *hsa-miR-150-5p* | 443.50980 | 787.28368 | 77.92440 | 340.26536 | 171.21829 | 1124.27140 | 525.40294 | 794.44000 | 320.28766 |
| *hsa-miR-24-2-5p* | 499.52391 | 629.96494 | 603.70587 | 427.28188 | 292.39906 | 288.03255 | 427.96265 | 868.32097 | 368.43916 |
| *hsa-miR-423-3p* | 278.33692 | 606.50513 | 393.86280 | 828.90926 | 330.85678 | 223.66184 | 366.32756 | 510.08608 | 1026.30741 |
| *hsa-miR-144-5p* | 1372.37559 | 757.61392 | 169.65029 | 238.63621 | 62.21935 | 273.36447 | 657.28309 | 38.72439 | 98.38335 |
| *hsa-miR-455-5p* | 202.68499 | 462.98628 | 938.51954 | 550.55528 | 490.33590 | 536.44018 | 151.74716 | 367.42884 | 670.61041 |
| *hsa-miR-182-5p* | 283.17912 | 218.72824 | 234.32224 | 1031.23366 | 1275.10707 | 51.07447 | 125.38785 | 912.45195 | 169.02867 |
| *hsa-miR-148a-3p* | 265.00592 | 893.54282 | 434.64248 | 280.88098 | 507.10361 | 464.99924 | 315.50368 | 418.00932 | 496.25084 |
| *hsa-miR-339-5p* | 203.34258 | 1260.61988 | 241.63002 | 166.12245 | 215.35968 | 300.95946 | 389.67756 | 544.63889 | 526.76268 |
| *hsa-miR-20a-5p* | 589.40354 | 295.31763 | 1181.11509 | 141.18211 | 274.76375 | 212.63440 | 412.47028 | 134.56107 | 589.43331 |
| *hsa-miR-99a-3p* | 535.87031 | 231.14814 | 277.37377 | 228.47330 | 675.54204 | 448.32617 | 415.84182 | 198.56197 | 968.10087 |
| *hsa-miR-149-5p* | 145.89374 | 580.28534 | 517.75427 | 393.00264 | 456.41096 | 273.25895 | 647.86507 | 334.96183 | 404.36858 |
| *hsa-miR-197-3p* | 354.85566 | 474.71619 | 294.20438 | 206.93891 | 175.87501 | 435.82137 | 351.86616 | 672.22902 | 608.28650 |
| *hsa-miR-335-5p* | 32.58025 | 106.25915 | 490.81393 | 319.82966 | 39.07743 | 606.24547 | 42.57614 | 441.69404 | 2002.42630 |
| *hsa-miR-214-5p* | 130.97856 | 322.22741 | 335.32484 | 77.95230 | 494.54996 | 502.30260 | 434.39922 | 572.24819 | 806.57014 |
| *hsa-miR-1260a* | 98.00975 | 1153.67074 | 19.63256 | 57.29686 | 57.36787 | 373.82499 | 222.88386 | 643.57681 | 474.66717 |
| *hsa-miR-409-3p* | 163.31969 | 62.09950 | 32.35491 | 147.88414 | 101.26137 | 688.71385 | 11.84219 | 2564.20763 | 270.27251 |
| *hsa-miR-542-3p* | 60.73675 | 607.19512 | 225.19698 | 310.21609 | 370.76640 | 148.31645 | 86.07179 | 558.85521 | 513.54377 |
| *hsa-miR-874-3p* | 119.11218 | 730.01414 | 181.29351 | 383.00453 | 349.44821 | 197.28040 | 170.61106 | 356.78032 | 208.72874 |
| *hsa-miR-193a-5p* | 139.10868 | 600.29518 | 224.81834 | 322.46652 | 215.50133 | 330.08456 | 52.30066 | 372.72566 | 394.18352 |
| *hsa-miR-421* | 208.45379 | 625.13498 | 276.74901 | 209.13630 | 204.04546 | 266.98016 | 91.64458 | 495.18364 | 171.41241 |
| *hsa-miR-328-3p* | 211.59225 | 282.20773 | 105.03513 | 360.31651 | 540.58591 | 191.63477 | 326.06413 | 437.24801 | 281.06434 |
| *hsa-miR-142-3p* | 555.71736 | 560.96550 | 125.06526 | 133.43632 | 58.62500 | 217.91069 | 326.70500 | 134.80807 | 206.99511 |
| *hsa-miR-99b-5p* | 239.06129 | 133.85893 | 675.98852 | 173.26395 | 348.91703 | 226.24722 | 112.59829 | 401.92676 | 397.21737 |
| *hsa-miR-365b-3p* | 227.61337 | 550.61558 | 365.71081 | 101.29953 | 234.21671 | 196.64725 | 172.22717 | 231.98954 | 342.69479 |
| *hsa-miR-196b-5p* | 144.10033 | 314.63747 | 183.66002 | 150.19139 | 410.85308 | 391.39503 | 332.58429 | 333.20538 | 273.69642 |
| *hsa-miR-146b-5p* | 835.72813 | 247.01802 | 82.39237 | 28.95057 | 91.39905 | 100.82986 | 82.72811 | 289.54095 | 311.92291 |
| *hsa-miR-671-5p* | 166.27881 | 510.59590 | 231.82321 | 287.91261 | 62.50264 | 58.98890 | 295.66454 | 278.26121 | 385.73209 |
| *hsa-miR-660-5p* | 165.80057 | 462.29629 | 229.96786 | 99.21202 | 152.66227 | 302.54234 | 98.61057 | 361.66547 | 330.55940 |
| *hsa-miR-590-5p* | 203.61159 | 531.98573 | 248.21838 | 63.06500 | 157.00027 | 109.06087 | 219.12223 | 183.13808 | 358.60082 |
| *hsa-miR-382-5p* | 60.10906 | 25.52979 | 14.86168 | 50.86951 | 20.91094 | 1002.75850 | 3.78950 | 1491.23197 | 163.82779 |
| *hsa-miR-92b-3p* | 190.37026 | 291.86766 | 214.00813 | 73.17298 | 150.71459 | 210.47112 | 385.74874 | 405.32989 | 361.50465 |
| *hsa-miR-23c* | 160.45024 | 536.81569 | 134.36091 | 74.60128 | 244.62792 | 302.85892 | 141.21458 | 269.09470 | 180.38393 |
| *hsa-miR-486-5p* | 585.30859 | 317.39745 | 62.45690 | 367.23828 | 21.60148 | 75.29263 | 339.94038 | 28.98154 | 40.22016 |
| *hsa-miR-134-5p* | 118.33504 | 13.10989 | 14.67235 | 91.57610 | 44.30074 | 756.46138 | 4.76474 | 1548.26194 | 111.12551 |
| *hsa-miR-379-5p* | 79.89633 | 33.11973 | 18.78061 | 62.35085 | 45.15064 | 777.56653 | 4.93192 | 1574.05990 | 110.99549 |
| *hsa-miR-505-3p* | 119.44097 | 374.66699 | 220.23678 | 91.52116 | 246.52247 | 199.17986 | 100.33814 | 313.11590 | 455.33723 |
| *hsa-miR-106b-3p* | 195.51136 | 487.13609 | 214.06492 | 122.28458 | 34.77484 | 152.90682 | 203.40695 | 363.69637 | 218.91380 |
| *hsa-miR-22-5p* | 178.53377 | 378.11696 | 180.36584 | 70.64599 | 189.04837 | 149.53000 | 253.42277 | 138.21121 | 526.67600 |
| *hsa-miR-16-2-3p* | 339.16334 | 396.74681 | 213.11832 | 122.11978 | 45.78806 | 85.10652 | 303.35499 | 149.60073 | 163.09100 |
| *hsa-miR-423-5p* | 177.78651 | 245.63803 | 188.27944 | 252.36988 | 192.30630 | 119.61344 | 155.53666 | 178.91161 | 470.28977 |
| *hsa-miR-146a-5p* | 125.29944 | 881.81292 | 50.15106 | 56.08830 | 16.23652 | 109.64126 | 116.61070 | 91.71999 | 95.65289 |
| *hsa-miR-128-3p* | 83.36358 | 244.25804 | 222.30037 | 209.13630 | 153.70693 | 100.46052 | 107.05336 | 197.21718 | 522.34193 |
| *hsa-miR-487b-3p* | 66.29632 | 25.52979 | 11.73788 | 46.41980 | 29.90566 | 502.98852 | 4.68115 | 1402.42112 | 83.34414 |
| *hsa-miR-20b-5p* | 422.88561 | 371.90701 | 83.05499 | 97.61891 | 31.88874 | 60.94112 | 263.45380 | 32.65912 | 58.16320 |
| *hsa-miR-337-3p* | 83.09457 | 26.90978 | 15.71362 | 53.94585 | 45.82347 | 611.62728 | 4.51396 | 1115.95396 | 141.59402 |
| *hsa-miR-99b-3p* | 58.52488 | 193.19845 | 528.90526 | 150.19139 | 87.46829 | 120.08831 | 85.79315 | 238.98793 | 277.12034 |
| *hsa-miR-30e-3p* | 166.03969 | 280.82774 | 94.54676 | 77.01841 | 283.31581 | 168.47187 | 167.21166 | 172.13278 | 203.52785 |
| *hsa-miR-135b-5p* | 4.48352 | 128.33897 | 505.82706 | 71.57988 | 165.90645 | 79.51366 | 15.77100 | 328.15556 | 519.52478 |
| *hsa-miR-489-3p* | 65.90774 | 924.59258 | 22.43450 | 11.64615 | 8.46353 | 50.01921 | 70.60729 | 20.33648 | 5.98101 |
| *hsa-miR-139-5p* | 100.31129 | 429.86655 | 73.15352 | 80.03982 | 62.44953 | 273.83934 | 46.72787 | 204.59979 | 299.39745 |
| *hsa-miR-324-5p* | 142.90473 | 371.90701 | 124.62982 | 106.73807 | 123.11073 | 129.48010 | 89.24828 | 275.37952 | 146.49151 |
| *hsa-miR-320b* | 128.01944 | 215.27827 | 221.22125 | 164.14480 | 61.52881 | 90.69939 | 182.73189 | 110.21768 | 437.87093 |
| *hsa-miR-4301* | 74.09764 | 155.93875 | 67.00060 | 110.47362 | 50.23230 | 71.22989 | 95.93563 | 806.51564 | 357.60399 |
| *hsa-miR-188-5p* | 113.58251 | 261.50790 | 196.13625 | 94.65244 | 91.84171 | 240.33491 | 68.29458 | 335.81262 | 177.09004 |
| *hsa-miR-196a-5p* | 33.83563 | 124.88900 | 180.04399 | 113.00062 | 331.88373 | 208.67718 | 88.05013 | 248.73077 | 361.59133 |
| *hsa-miR-27b-5p* | 201.51928 | 103.49917 | 93.35404 | 166.56192 | 253.26851 | 198.01908 | 220.93338 | 119.54885 | 199.41049 |
| *hsa-miR-155-5p* | 139.07879 | 162.83869 | 147.67015 | 16.31560 | 104.50159 | 127.68616 | 84.76218 | 210.93950 | 498.07114 |
| *hsa-miR-18a-5p* | 173.09376 | 253.22797 | 343.92000 | 40.54179 | 25.12500 | 107.58351 | 137.59226 | 86.28595 | 119.40359 |
| *hsa-miR-374a-5p* | 189.53334 | 238.73808 | 113.27058 | 53.50637 | 132.45957 | 177.17774 | 101.64775 | 99.87105 | 206.30166 |
| *hsa-miR-21-3p* | 97.56140 | 171.11863 | 248.40770 | 40.37698 | 54.35782 | 136.39204 | 160.91440 | 219.72179 | 317.99060 |
| *hsa-miR-326* | 218.25775 | 323.60740 | 70.84381 | 74.87596 | 51.63108 | 146.57528 | 79.91385 | 129.04470 | 126.64148 |
| *hsa-miR-454-3p* | 114.44932 | 204.23836 | 261.67908 | 130.14024 | 89.80550 | 147.63053 | 86.62907 | 73.16741 | 191.47914 |
| *hsa-miR-125b-2-3p* | 183.58520 | 73.82941 | 70.20012 | 82.12733 | 193.19160 | 264.81688 | 99.30717 | 72.34407 | 301.26110 |
| *hsa-miR-127-3p* | 43.87872 | 24.14981 | 7.93254 | 30.59861 | 21.45983 | 294.25857 | 2.31271 | 1079.04092 | 66.96136 |
| *hsa-miR-664a-3p* | 60.55741 | 144.89884 | 60.71515 | 62.35085 | 186.33933 | 146.10041 | 140.26720 | 306.72130 | 239.76067 |
| *hsa-miR-29c-5p* | 259.71537 | 150.41879 | 57.19379 | 92.23531 | 141.06475 | 151.58775 | 29.08998 | 104.31708 | 148.74523 |
| *hsa-miR-204-5p* | 22.50727 | 416.06666 | 48.25785 | 2.91154 | 12.07558 | 36.30086 | 25.52339 | 607.26755 | 6.97785 |
| *hsa-miR-337-5p* | 34.70244 | 23.45981 | 16.60343 | 40.32205 | 29.40988 | 536.70399 | 3.09290 | 700.68911 | 93.44252 |
| *hsa-miR-136-5p* | 62.29104 | 15.86987 | 9.97720 | 26.86305 | 28.27669 | 303.06997 | 3.12076 | 916.67843 | 92.44568 |
| *hsa-miR-193a-3p* | 103.30030 | 136.61890 | 135.57256 | 62.90020 | 59.61655 | 169.15779 | 136.95139 | 103.90541 | 177.22006 |
| *hsa-miR-17-3p* | 86.71128 | 156.62874 | 204.16345 | 34.88352 | 66.87606 | 92.80990 | 74.78688 | 131.35005 | 183.72116 |
| *hsa-miR-744-5p* | 90.11875 | 186.29850 | 92.97540 | 120.96615 | 112.04440 | 72.54896 | 56.95395 | 147.98150 | 106.48806 |
| *hsa-miR-206* | 1.16572 | 10.34992 | 2.87767 | 224.57294 | 246.96512 | 23.79606 | 3.23222 | 637.70366 | 3.03385 |
| *hsa-miR-340-3p* | 111.28097 | 219.41824 | 56.96660 | 43.06878 | 109.24683 | 70.91331 | 65.56391 | 160.52370 | 76.32295 |
| *hsa-let-7b-3p* | 93.31700 | 140.06888 | 79.43897 | 59.76892 | 83.39588 | 82.46838 | 127.83987 | 167.79653 | 153.77275 |
| *hsa-miR-27a-5p* | 58.94334 | 326.36738 | 29.85588 | 74.93089 | 3.78911 | 70.70226 | 153.22395 | 51.87037 | 100.46371 |
| *hsa-miR-494-3p* | 68.23917 | 30.35976 | 10.50730 | 51.36392 | 37.80259 | 169.84370 | 4.37464 | 632.65385 | 154.59622 |
| *hsa-miR-3613-5p* | 144.45901 | 193.88844 | 49.84814 | 69.65716 | 145.82770 | 23.47948 | 45.25108 | 16.63145 | 170.80564 |
| *hsa-miR-138-1-3p* | 11.38814 | 106.25915 | 157.83667 | 382.12558 | 33.11047 | 28.96682 | 101.67561 | 66.33370 | 53.87247 |
| *hsa-miR-495-3p* | 57.14994 | 33.80973 | 11.32138 | 23.18243 | 21.12342 | 235.79730 | 2.22912 | 622.69144 | 115.58961 |
| *hsa-let-7d-3p* | 64.32357 | 143.51885 | 132.50556 | 91.79584 | 92.49683 | 57.66983 | 61.57936 | 116.85928 | 140.59718 |
| *hsa-miR-24-1-5p* | 98.06953 | 102.11918 | 74.72488 | 69.21769 | 173.85651 | 106.00062 | 92.20186 | 95.04079 | 97.21315 |
| *hsa-let-7i-3p* | 29.53145 | 87.62930 | 81.93800 | 76.52400 | 203.92152 | 60.99389 | 74.25747 | 82.58092 | 257.14028 |
| *hsa-miR-15b-3p* | 143.74165 | 164.90868 | 102.95260 | 40.98127 | 15.03251 | 43.00175 | 140.68516 | 66.99237 | 75.80286 |
| *hsa-miR-183-5p* | 33.74596 | 40.70967 | 71.65789 | 199.08325 | 246.31000 | 19.31121 | 18.22303 | 208.90860 | 37.87976 |
| *hsa-miR-1306-5p* | 30.60750 | 162.14870 | 92.95647 | 21.31465 | 141.75529 | 73.60421 | 47.67525 | 123.88511 | 92.27232 |
| *hsa-miR-339-3p* | 37.27300 | 178.01857 | 42.40784 | 54.16559 | 76.96855 | 41.20781 | 71.22030 | 159.42592 | 97.73324 |
| *hsa-miR-503-5p* | 18.71122 | 123.50901 | 175.23525 | 89.70832 | 41.85730 | 34.92903 | 21.48312 | 50.66281 | 228.36207 |
| *hsa-miR-195-3p* | 66.68489 | 85.55931 | 43.78988 | 100.09097 | 93.59461 | 76.71722 | 161.33236 | 56.34385 | 88.37166 |
| *hsa-miR-193b-5p* | 56.31301 | 111.08911 | 47.15979 | 204.02737 | 54.10994 | 77.45590 | 31.68133 | 98.08714 | 48.54157 |
| *hsa-miR-502-3p* | 63.75565 | 132.47894 | 69.10206 | 44.60695 | 48.97516 | 80.35786 | 42.26964 | 113.42870 | 122.65414 |
| *hsa-miR-590-3p* | 70.95918 | 97.97921 | 104.82688 | 28.56603 | 64.11390 | 31.60496 | 77.07173 | 83.02004 | 168.85531 |
| *hsa-miR-424-3p* | 9.98330 | 125.57899 | 121.43030 | 101.02486 | 53.29546 | 32.55470 | 71.97262 | 75.08854 | 125.60130 |
| *hsa-miR-323a-3p* | 22.05892 | 17.93986 | 5.98253 | 10.71226 | 11.96935 | 268.98515 | 1.75543 | 522.54595 | 28.17144 |
| *hsa-miR-154-5p* | 27.14024 | 1.37999 | 7.93254 | 10.98693 | 17.70613 | 517.92041 | 1.17029 | 240.16805 | 51.66210 |
| *hsa-miR-324-3p* | 90.92579 | 139.37888 | 57.98893 | 28.56603 | 27.94028 | 32.97680 | 7.85764 | 130.44438 | 124.04104 |
| *hsa-miR-376a-5p* | 26.15387 | 13.10989 | 4.99807 | 16.53534 | 11.49128 | 429.38430 | 1.42106 | 317.47960 | 36.57954 |
| *hsa-miR-628-5p* | 71.28797 | 84.17932 | 33.90734 | 35.32299 | 112.91200 | 52.81564 | 73.89524 | 169.55299 | 52.31221 |
| *hsa-miR-539-5p* | 16.70858 | 6.20995 | 6.13399 | 26.20384 | 11.72146 | 243.81726 | 2.36844 | 521.53050 | 29.68837 |
| *hsa-miR-766-3p* | 43.04179 | 80.03936 | 149.54443 | 82.01746 | 41.69794 | 67.27267 | 25.10543 | 137.52509 | 58.68329 |
| *hsa-miR-296-5p* | 2.45099 | 24.14981 | 239.54749 | 60.81268 | 130.68896 | 71.81028 | 69.29768 | 28.59732 | 71.29543 |
| *hsa-miR-362-5p* | 49.61762 | 97.97921 | 82.69528 | 30.48874 | 51.59567 | 93.07372 | 35.77733 | 124.04977 | 92.27232 |
| *hsa-miR-299-5p* | 22.80617 | 17.24986 | 7.51603 | 13.56886 | 9.10095 | 261.86216 | 1.47679 | 406.86679 | 58.85665 |
| *hsa-miR-330-3p* | 78.07303 | 114.53908 | 103.21765 | 49.38627 | 37.89112 | 42.15754 | 32.76802 | 83.73360 | 46.98130 |
| *hsa-miR-329-3p* | 26.24354 | 11.03991 | 6.39904 | 13.34913 | 11.24339 | 167.68043 | 2.98144 | 513.21477 | 43.90411 |
| *hsa-miR-4284* | 47.94377 | 236.66810 | 15.03206 | 5.49347 | 8.53436 | 74.39566 | 61.91373 | 5.92804 | 44.16416 |
| *hsa-miR-425-3p* | 32.84926 | 124.88900 | 86.38704 | 38.12466 | 63.90143 | 52.18249 | 24.35311 | 69.76428 | 54.04583 |
| *hsa-miR-96-5p* | 24.92837 | 18.62985 | 44.50930 | 95.25672 | 254.27776 | 5.59287 | 8.44278 | 119.08229 | 20.67351 |
| *hsa-miR-542-5p* | 9.44528 | 135.92891 | 35.08113 | 70.92066 | 78.49128 | 63.84308 | 14.62858 | 104.15241 | 44.25084 |
| *hsa-miR-132-3p* | 43.31080 | 77.96937 | 64.06613 | 11.86589 | 35.42997 | 75.71473 | 58.62578 | 114.38926 | 95.00278 |
| *hsa-miR-323b-3p* | 13.92880 | 13.79989 | 3.52137 | 6.64710 | 10.18103 | 210.73493 | 2.67494 | 431.31997 | 4.98418 |
| *hsa-miR-130b-5p* | 42.50377 | 66.92946 | 65.71322 | 24.06139 | 53.66729 | 78.66945 | 42.91051 | 80.79702 | 113.98600 |
| *hsa-miR-935* | 19.66771 | 99.35920 | 28.18985 | 5.87801 | 61.06845 | 4.43208 | 33.77113 | 196.61340 | 119.83699 |
| *hsa-miR-362-3p* | 38.43871 | 113.84909 | 77.75401 | 15.49158 | 46.70878 | 72.60172 | 23.18282 | 51.54103 | 75.62949 |
| *hsa-miR-194-5p* | 63.39697 | 89.00929 | 50.37824 | 50.64977 | 41.25529 | 30.65523 | 36.80830 | 78.68378 | 25.00757 |
| *hsa-miR-23a-5p* | 22.59694 | 92.45926 | 14.59663 | 45.15630 | 6.55127 | 58.88337 | 142.77496 | 28.32287 | 113.42257 |
| *hsa-miR-671-3p* | 23.76266 | 90.38927 | 68.62876 | 24.72060 | 27.62157 | 33.92653 | 66.12119 | 111.20568 | 57.68645 |
| *hsa-miR-7-1-3p* | 43.22113 | 103.49917 | 39.88987 | 14.11821 | 56.65962 | 42.10478 | 47.98175 | 29.39321 | 75.15275 |
| *hsa-miR-940* | 31.17541 | 134.54892 | 76.92100 | 29.60979 | 19.52986 | 17.20070 | 28.19833 | 40.67296 | 39.26666 |
| *hsa-miR-181d-5p* | 28.63475 | 126.26899 | 35.25152 | 11.42641 | 67.76137 | 26.64525 | 30.45531 | 36.06226 | 53.22236 |
| *hsa-miR-532-3p* | 23.58332 | 102.80917 | 29.70442 | 8.84448 | 35.96115 | 46.58962 | 22.51408 | 77.17433 | 69.77850 |
| *hsa-miR-450a-5p* | 8.07034 | 79.34936 | 50.94620 | 35.15819 | 63.08695 | 13.56006 | 1.50465 | 70.53273 | 102.19733 |
| *hsa-miR-93-3p* | 34.28398 | 63.47949 | 79.51470 | 25.32488 | 28.22357 | 32.87127 | 35.80520 | 70.28572 | 47.76143 |
| *hsa-miR-485-3p* | 22.47738 | 6.20995 | 5.05486 | 21.14985 | 8.18023 | 126.84196 | 1.81116 | 271.92150 | 29.60169 |
| *hsa-miR-32-5p* | 45.82157 | 54.50956 | 54.31611 | 14.61262 | 39.05973 | 50.17750 | 42.26964 | 35.73292 | 47.58807 |
| *hsa-miR-598-3p* | 9.02682 | 108.32913 | 102.70648 | 21.91893 | 11.33192 | 13.98216 | 25.02184 | 20.83048 | 41.30367 |
| *hsa-miR-874-5p* | 20.20573 | 86.24931 | 26.31558 | 60.92255 | 35.14667 | 32.29088 | 24.43670 | 49.07102 | 30.16512 |
| *hsa-miR-369-3p* | 22.65672 | 10.34992 | 6.91021 | 7.19644 | 12.85465 | 116.18386 | 1.69970 | 244.22985 | 42.21383 |
| *hsa-miR-675-3p* | 45.88135 | 20.00984 | 2.06360 | 13.62380 | 127.20085 | 28.06985 | 18.97536 | 23.98662 | 99.51021 |
| *hsa-let-7f-1-3p* | 32.61014 | 55.88955 | 46.04280 | 29.06044 | 22.84091 | 39.09729 | 35.35937 | 60.24099 | 47.41471 |
| *hsa-miR-26a-5p* | 21.84969 | 37.25970 | 32.80928 | 22.02880 | 45.84117 | 46.43133 | 25.88562 | 78.40934 | 64.92435 |
| *hsa-miR-29b-1-5p* | 38.91695 | 51.74958 | 16.69809 | 44.38721 | 22.16808 | 20.63029 | 51.24183 | 52.14482 | 48.88829 |
| *hsa-miR-501-5p* | 24.33057 | 84.86932 | 34.81608 | 18.62285 | 23.61998 | 32.71298 | 11.42423 | 56.06940 | 40.17681 |
| *hsa-miR-152-5p* | 32.58025 | 44.84964 | 30.06413 | 60.81268 | 48.28462 | 33.45166 | 23.15496 | 31.34178 | 31.81206 |
| *hsa-miR-221-5p* | 71.67654 | 32.42974 | 31.71122 | 5.93294 | 12.53594 | 39.20282 | 26.55436 | 60.10376 | 49.75511 |
| *hsa-miR-34a-3p* | 54.75872 | 6.89994 | 20.67382 | 21.09491 | 53.49022 | 68.75003 | 17.94439 | 68.47438 | 44.51088 |
| *hsa-miR-132-5p* | 29.71079 | 52.43958 | 31.40831 | 10.43759 | 18.99868 | 28.38643 | 47.56379 | 62.60122 | 60.72030 |
| *hsa-miR-1307-5p* | 24.71914 | 83.48933 | 39.45444 | 9.99811 | 39.64403 | 18.83635 | 15.13013 | 27.33486 | 47.89146 |
| *hsa-miR-505-5p* | 20.80353 | 31.73975 | 23.79761 | 33.01574 | 49.59487 | 32.66022 | 19.89487 | 81.64780 | 62.06386 |
| *hsa-miR-210-5p* | 23.55343 | 11.72991 | 10.37477 | 20.98504 | 131.52115 | 14.19321 | 85.15228 | 21.21471 | 32.93892 |
| *hsa-miR-106a-5p* | *94.63216* | *62.09950* | *27.01606* | *9.77837* | *6.10862* | *9.91942* | *41.29440* | *5.54382* | *13.52229* |
| *hsa-miR-1247-5p* | 22.77628 | 18.62985 | 2.63156 | 82.12733 | 20.57453 | 51.28552 | 1.53252 | 87.98752 | 75.71618 |
| *hsa-miR-369-5p* | 17.12705 | 8.27993 | 3.02913 | 5.82308 | 10.74762 | 134.54534 | 0.94737 | 186.29422 | 23.31729 |
| *hsa-miR-769-5p* | 10.82023 | 84.86932 | 32.65782 | 10.54746 | 32.33140 | 20.84134 | 24.29738 | 5.81826 | 77.79653 |
| *hsa-miR-18b-5p* | 44.80531 | 77.27938 | 49.05300 | 7.36125 | 6.17944 | 19.68055 | 31.68133 | 11.33464 | 26.09109 |
| *hsa-miR-26b-3p* | 30.96618 | 23.45981 | 23.70295 | 16.59027 | 36.24445 | 66.74504 | 41.93527 | 38.50483 | 48.45489 |
| *hsa-miR-877-5p* | 27.02068 | 47.60962 | 44.16852 | 12.63497 | 22.09725 | 44.58463 | 8.74929 | 33.23546 | 60.07019 |
| *hsa-miR-642a-5p* | 8.04045 | 114.53908 | 5.60389 | 4.28490 | 32.11892 | 11.55507 | 51.43688 | 24.45317 | 9.96836 |
| *hsa-miR-29b-2-5p* | 47.76443 | 41.39967 | 14.93740 | 19.06233 | 31.21591 | 29.86379 | 19.14254 | 37.35216 | 45.46438 |
| *hsa-miR-642a-3p* | 16.67869 | 99.35920 | 8.95487 | 15.16197 | 32.49075 | 16.83136 | 41.26653 | 23.02605 | 4.59411 |
| *hsa-miR-1260b* | 12.31473 | 104.18916 | 1.89321 | 8.40500 | 7.22410 | 36.40639 | 13.98771 | 33.40013 | 49.23502 |
| *hsa-miR-31-3p* | 3.28791 | 4.13997 | 102.82008 | 0.54935 | 12.57135 | 7.91443 | 141.79972 | 42.53919 | 24.92089 |
| *hsa-miR-29a-5p* | 28.45541 | 41.39967 | 34.62676 | 7.47112 | 27.67468 | 16.09268 | 32.43366 | 25.24907 | 67.43810 |
| *hsa-miR-1301-3p* | 25.64573 | 65.54947 | 14.23692 | 12.14056 | 20.00793 | 16.25097 | 11.81432 | 28.56987 | 66.74465 |
| *hsa-miR-15a-3p* | 18.95034 | 60.71951 | 50.49183 | 12.68991 | 11.11945 | 32.07983 | 20.56361 | 16.79612 | 39.00662 |
| *hsa-miR-7-5p* | 45.91124 | 51.74958 | 28.49277 | 21.03998 | 14.74921 | 13.29625 | 17.94439 | 9.16651 | 43.60073 |
| *hsa-miR-181a-2-3p* | 8.18990 | 71.75942 | 22.07479 | 71.68975 | 2.53198 | 15.19571 | 7.60686 | 43.08809 | 10.14172 |
| *hsa-miR-452-5p* | 12.55386 | 53.81957 | 17.00100 | 1.64804 | 25.24894 | 19.52226 | 7.91337 | 27.52697 | 117.97334 |
| *hsa-miR-301b-3p* | 16.26023 | 39.32968 | 40.72288 | 19.44687 | 28.54228 | 41.68267 | 8.69356 | 46.93033 | 30.98859 |
| *hsa-miR-1307-3p* | 18.98023 | 80.72935 | 22.77528 | 8.18527 | 26.34672 | 12.82138 | 12.17655 | 25.74307 | 27.17461 |
| *hsa-miR-190a-5p* | 43.40047 | 31.04975 | 23.34324 | 16.75507 | 41.64482 | 12.76862 | 28.69988 | 14.60055 | 30.46850 |
| *hsa-miR-363-3p* | 72.03522 | 44.15965 | 12.43837 | 14.77743 | 2.17785 | 10.13047 | 34.66277 | 18.19580 | 9.49161 |
| *hsa-miR-26a-2-3p* | 20.08617 | 26.90978 | 21.88547 | 40.81646 | 27.33827 | 29.70550 | 22.54195 | 32.05534 | 33.37233 |
| *hsa-miR-497-3p* | 34.37365 | 35.18972 | 26.95927 | 12.90965 | 13.88161 | 18.46701 | 55.19852 | 6.75138 | 40.43686 |
| *hsa-miR-551b-3p* | 8.27957 | 68.30945 | 5.98253 | 12.25043 | 19.17574 | 45.27055 | 8.94433 | 21.84593 | 52.26887 |
| *hsa-miR-4315* | 4.75253 | 57.95953 | 6.47477 | 86.63198 | 8.44582 | 11.34402 | 6.63162 | 21.40682 | 28.43149 |
| *hsa-miR-24-3p* | 30.18903 | 21.38983 | 18.83741 | 22.08374 | 31.33985 | 30.33865 | 37.53276 | 35.37614 | 25.61434 |
| *hsa-miR-145-3p* | 12.07561 | 31.73975 | 24.59276 | 8.89942 | 17.97172 | 70.06910 | 15.32518 | 45.47577 | 40.39352 |
| *hsa-miR-215-5p* | 34.70244 | 15.86987 | 3.06700 | 100.14591 | 17.67072 | 13.50730 | 5.09911 | 24.91973 | 11.05187 |
| *hsa-miR-550a-5p* | 19.51826 | 51.74958 | 22.05586 | 25.05021 | 14.74921 | 13.98216 | 25.32834 | 21.05004 | 15.08256 |
| *hsa-miR-331-5p* | 17.51562 | 44.84964 | 11.30245 | 31.97198 | 16.02405 | 22.10765 | 14.04344 | 42.23730 | 17.37961 |
| *hsa-miR-199a-3p* | 6.78506 | 25.52979 | 13.70682 | 6.64710 | 32.04810 | 48.54185 | 17.91653 | 21.37937 | 70.51529 |
| *hsa-miR-125a-3p* | 11.38814 | 27.59978 | 39.98453 | 27.41240 | 11.82770 | 20.41923 | 15.21373 | 34.16858 | 36.36283 |
| *hsa-miR-500a-3p* | 14.37715 | 53.81957 | 17.94760 | 11.37148 | 13.68684 | 19.99713 | 9.83598 | 33.07079 | 27.39131 |
| *hsa-miR-1226-3p* | 9.32572 | 19.31984 | 63.89574 | 14.39288 | 18.36126 | 18.25596 | 5.65639 | 31.94556 | 33.58903 |
| *hsa-miR-450b-5p* | 4.48352 | 33.11973 | 23.81654 | 23.95152 | 33.71247 | 12.76862 | 6.21366 | 30.21655 | 39.57004 |
| *hsa-miR-143-5p* | 11.11913 | 16.55987 | 19.04566 | 21.25972 | 21.47754 | 49.43882 | 11.06199 | 29.31088 | 36.79624 |
| *hsa-miR-615-3p* | 6.00792 | 49.67960 | 20.65489 | 5.87801 | 20.30893 | 6.91194 | 8.63783 | 21.29704 | 50.70860 |
| *hsa-miR-133a-3p* | 2.27165 | 6.89994 | 11.43497 | 32.46639 | 37.51929 | 16.35649 | 3.42727 | 98.30670 | 10.18506 |
| *hsa-miR-500b-5p* | 17.18683 | 38.63969 | 27.96267 | 8.89942 | 12.39429 | 22.37146 | 6.93813 | 22.55949 | 24.92089 |
| *hsa-miR-148b-5p* | 11.68704 | 34.49972 | 33.01753 | 15.71132 | 19.06950 | 13.92940 | 11.06199 | 26.67619 | 18.20309 |
| *hsa-miR-103a-2-5p* | 19.84705 | 37.25970 | 16.54663 | 6.97670 | 16.20111 | 20.63029 | 10.69976 | 19.89736 | 31.42200 |
| *hsa-miR-1296-5p* | 7.65187 | 34.49972 | 22.09373 | 6.09775 | 36.27986 | 17.20070 | 15.01868 | 12.18542 | 33.28565 |
| *hsa-miR-454-5p* | 14.58639 | 24.14981 | 43.92240 | 13.56886 | 14.20032 | 23.42672 | 8.72142 | 22.42227 | 18.02972 |
| *hsa-miR-187-3p* | 13.60001 | 31.73975 | 9.63642 | 18.34818 | 34.96961 | 16.19820 | 4.84833 | 12.84409 | 33.76239 |
| *hsa-miR-98-3p* | 9.47517 | 36.56971 | 21.16606 | 10.49252 | 13.86390 | 13.82387 | 6.07434 | 29.61277 | 26.17777 |
| *hsa-miR-627-5p* | 19.30903 | 26.21979 | 19.02673 | 25.98410 | 17.61760 | 4.90695 | 10.36540 | 14.07910 | 15.77601 |
| *hsa-miR-504-5p* | 5.82858 | 14.48988 | 12.36264 | 7.47112 | 7.02933 | 22.16041 | 2.78640 | 101.79217 | 14.69249 |
| *hsa-miR-153-3p* | 4.06506 | 22.76982 | 5.28205 | 7.47112 | 39.59091 | 14.50979 | 1.56038 | 22.72416 | 43.68741 |
| *hsa-miR-33a-5p* | 6.21715 | 11.72991 | 23.32431 | 6.42736 | 21.79625 | 23.42672 | 13.59762 | 20.61092 | 31.03193 |
| *hsa-miR-338-5p* | 20.53452 | 22.07982 | 19.23498 | 10.27278 | 3.96617 | 18.88911 | 11.03413 | 12.24031 | 15.90603 |
| *hsa-miR-744-3p* | 9.47517 | 22.07982 | 23.83548 | 10.98693 | 9.91543 | 15.35400 | 9.05579 | 18.14091 | 15.21258 |
| *hsa-miR-10a-3p* | 7.23341 | 35.87971 | 15.22138 | 6.26255 | 5.15248 | 12.76862 | 3.03717 | 12.26775 | 30.29514 |
| *hsa-let-7e-3p* | 7.95078 | 8.96993 | 31.37044 | 9.11916 | 10.28726 | 10.44705 | 10.86695 | 30.02444 | 25.96107 |
| *hsa-miR-629-5p* | 11.89627 | 26.90978 | 23.64616 | 6.15268 | 3.08087 | 10.39429 | 6.13007 | 13.53021 | 23.88072 |
| *hsa-miR-769-3p* | 4.99165 | 38.63969 | 12.40051 | 8.02046 | 9.33113 | 11.39678 | 6.46444 | 10.18196 | 19.67667 |
| *hsa-miR-23b-5p* | 28.45541 | 10.34992 | 5.20632 | 12.08563 | 17.29889 | 10.60534 | 16.30042 | 1.64668 | 19.37329 |
| *hsa-miR-10b-3p* | 9.11649 | 20.00984 | 11.81361 | 9.33889 | 11.36734 | 27.85880 | 12.45519 | 23.05350 | 5.63429 |
| *hsa-miR-589-3p* | 10.22243 | 18.62985 | 23.04033 | 15.76625 | 5.86073 | 6.01497 | 8.97220 | 17.01568 | 20.19676 |
| *hsa-miR-2110* | 19.93672 | 14.48988 | 5.73642 | 8.35007 | 13.49207 | 9.60284 | 9.66880 | 21.02259 | 21.75702 |
| *hsa-let-7c-3p* | 18.05364 | 9.65992 | 6.30438 | 6.59216 | 11.75687 | 34.92903 | 13.40257 | 8.15106 | 11.91869 |
| *hsa-miR-502-5p* | 9.65451 | 14.48988 | 14.14226 | 6.15268 | 7.66676 | 13.34901 | 3.59445 | 16.57656 | 22.36379 |
| *hsa-miR-101-5p* | 16.05100 | 12.41990 | 3.69175 | 4.77932 | 13.75766 | 16.46202 | 10.44899 | 13.99677 | 12.87218 |
| *hsa-miR-20a-3p* | 7.05407 | 11.72991 | 30.93500 | 3.73556 | 8.83536 | 11.87165 | 7.41181 | 10.70341 | 12.65548 |
| *hsa-miR-3940-3p* | 5.29055 | 12.41990 | 10.26118 | 5.54840 | 20.89324 | 8.65311 | 6.54803 | 17.94879 | 14.69249 |
| *hsa-miR-491-5p* | 12.58375 | 11.03991 | 10.99953 | 10.65733 | 8.55206 | 12.55756 | 10.53258 | 9.33118 | 11.18190 |
