# Supplementary material for: Expression profiling of microRNAs and isomiRs in conventional central chondrosarcoma
Source: Cell Death Discov. 2020 Jun 10;6:46. doi: 10.1038/s41420-020-0282-3 (PMC7287106; doi:10.1038/s41420-020-0282-3)
Supplement: Supplementary file 5 — Table S2B [file 41420_2020_282_MOESM5_ESM.docx]

**Supplementary Table 2B**

IsomiR ID in the shortened form (ShortID)

| This is an example of ID in the shortened form (ShortID). |
| --- |
| **hsa-miR-493-3p.3.P0.S.0** |
|  |
| The four fields separated by the dot character represent respectively: |
| **1) the known microRNA to which the isomiR refers** |
| **2) the type of isomiR, indicated by one of the following values:** |
| **• 5 for 5'-isomiRs** |
| **• 3 for 3'-isomiRs** |
| **• 53 for isomiRs having a shift both at the 5' and the 3'** |
| **• 0 for the isomiRs with no shift compared to the known form** |
| **3) the number of mismatches compared to the precursor sequence, in the region corresponding to the mature sequence (P0: 0 mismatch; P1: 1 mismatch)** |
| **4) the possibility that the isomiRs maps to more than one known precursor (S: single mapping; M: multiple mappings)** |
| **5) a numeric counter useful to discriminate between isomiRs with the same features** |
