# Supplementary material for: Expression profiling of microRNAs and isomiRs in conventional central chondrosarcoma
Source: Cell Death Discov. 2020 Jun 10;6:46. doi: 10.1038/s41420-020-0282-3 (PMC7287106; doi:10.1038/s41420-020-0282-3)
Supplement: Supplementary file 5 [file 41420_2020_282_MOESM4_ESM.docx]

Table S2A. List of the isomiRs of the differently modulated miRNAs.

Mature miRNA	isomiR short ID	GI		GII			GIII				isomiR sequence	sequence variation		
		Pt1	Pt2	Pt3	Pt4	Pt5	Pt7	Pt6	Pt8	Pt9		***5'-end shift***	***3'-end shift***	***mismatches***
hsa-miR-134-5p	*hsa-miR-134-5p.0.P1.S.25*	27.7804437	0.0000000	4.631901627	15.7166791	9.093225365	1.067941088	169.7828412	190.1755075	1.345828101	tgtgactggttgaccagaggga			P1:29.g>a
	hsa-miR-134-5p.3.P0.S.17	21.77909349	2.747664142	3.279731587	14.39471544	3.853061595	0.512611722	88.26657017	196.3858833	22.65477304	tgtgactggttgaccagaggg		3t:-g	P0:
hsa-miR-136-5p	*hsa-miR-136-5p.3.P0.S.3*	44.09056483	10.99065657	7.192393831	15.7166791	8.528109664	2.221317463	167.9884578	699.1963084	75.14206898	actccatttgttttgatgatgg		3t:-a	P0:
hsa-miR-142-5p	*hsa-miR-142-5p.53.P0.S.42*	3726.499693	2790.252936	331.339199	1098.037707	211.3789591	1543.815636	1073.468462	416.9692308	325.8399369	cccataaagtagaaagcact	5t:+cc	3t:-act	P0:
	hsa-miR-142-5p.53.P0.S.44	487.8516942	414.8972854	17.98098458	54.86149199	13.53708974	186.4625139	80.23456862	30.86786781	20.9351038	cccataaagtagaaagca	5t:+cc	3t:-ctact	P0:
	hsa-miR-142-5p.53.P0.S.45	237.488915	309.112216	33.5740944	75.35192876	19.49649167	126.8714012	99.37465741	62.51778295	44.8609367	cccataaagtagaaagcacta	5t:+cc	3t:-ct	P0:
	hsa-miR-142-5p.53.P0.S.46	133.868828	153.869192	6.818389351	36.79465527	5.830966547	63.60657119	43.91966802	20.88526377	14.05642683	cccataaagtagaaagcac	5t:+cc	3t:-tact	P0:
	hsa-miR-142-5p.53.P0.S.47	192.5755845	197.8318182	49.82890446	60.81032847	21.88538986	86.5886634	89.97550667	81.65494094	80.89922252	cccataaagtagaaagcactac	5t:+cc	3t:-t	P0:
hsa-miR-144-3p	*hsa-miR-144-3p.3.P0.S.12*	159.3745664	81.05609219	26.12277439	7.564569846	0.66786401	94.06425101	14.95319437	7.72846765	20.9351038	tacagtatagatgatgtac		3t:-t	P0:
	hsa-miR-144-3p.5.P0.S.5	209.4180834	100.2897412	39.52939649	20.63732162	0.642176933	143.2749763	8.202895195	9.062548375	10.99092949	ctacagtatagatgatgtact	5t:+c		P0:
hsa-miR-144-5p	*hsa-miR-144-5p.3.P0.S.13*	1009.29159	266.5234218	104.6924846	146.2238696	38.35080641	425.8949058	206.1831886	25.62355048	56.00140265	ggatatcatcatatactgtaagt		3t:+t	P0:
hsa-miR-146a-5p	*hsa-miR-146a-5p.3.P0.S.0*	19.45599019	174.476673	9.091185802	5.214412224	2.106340339	16.91618683	23.32698321	3.312200421	22.05662721	tgagaactgaattccatgggtta		3n:+a	P0:
	hsa-miR-146a-5p.3.P0.S.16	24.19899277	160.7383523	9.263803254	11.97111539	1.721034179	20.97436296	14.18417294	11.08667085	15.47702316	tgagaactgaattccatgggttg		3t:+g	P0:
	hsa-miR-146a-5p.3.P0.S.22	13.11585408	118.1495581	6.358076146	6.389491035	1.643972947	9.995928581	23.83966416	11.54669869	11.2900024	tgagaactgaattccatgggt		3t:-t	P0:
hsa-miR-20b-5p	*hsa-miR-20b-5p.3.P0.S.7*	146.2587123	107.1589015	23.67736049	20.41699434	4.109932368	79.71112279	17.00391816	4.048244959	11.06569772	caaagtgctcatagtgcaggt		3t:-ag	P0:
	hsa-miR-20b-5p.3.P0.S.9	167.8926118	63.19627527	19.01668929	21.07797617	4.1869936	90.5186866	18.20017371	5.014303415	13.30874456	caaagtgctcatagtgcaggta		3t:-g	P0:
hsa-miR-31-5p	*hsa-miR-31-5p.0.P1.S.47*	6.824115961	8.242992426	190.6847452	0.440654554	8.271238891	60.91535965	19.14008879	275.5106712	39.85146544	aggcaagatgctggcatagca			P1:28.t>a
	hsa-miR-31-5p.0.P1.S.49	5.614166323	8.242992426	114.4166011	0.734424257	14.23064083	126.9141189	26.40306891	45.12873074	20.33695797	aggcaagatgctggcatagcg			P1:28.t>g
	hsa-miR-31-5p.3.P0.S.0	5.904554236	9.616824497	206.6806291	0.146884851	5.419973311	13.8405165	11.96255549	360.3398042	67.96431911	aggcaagatgctggcatagcta		3n:+a	P0:
	hsa-miR-31-5p.3.P0.S.32	97.47354288	64.57010734	1236.286191	5.214412224	51.70808661	317.306656	98.86197646	2414.594107	623.2679473	aggcaagatgctggcatagctg		3t:+g	P0:
	hsa-miR-31-5p.3.P0.S.33	183.0895793	37.09346592	1340.978675	1.836060642	215.3090819	24.94710381	179.609226	2583.332317	132.264995	aggcaagatgctggcatagctgt		3t:+gt	P0:
	hsa-miR-31-5p.3.P0.S.42	9.050423296	9.616824497	161.0808522	0.146884851	19.72767537	430.3802584	39.3909863	317.3732043	67.74001442	aggcaagatgctggcatagc		3t:-t	P0:
	hsa-miR-31-5p.3.P0.S.6	74.24250982	37.09346592	994.9094538	3.598678859	63.16452308	11.61919903	121.0781509	2317.252217	207.1827593	aggcaagatgctggcatagctga		3n:+ga	P0:
hsa-miR-335-5p	*hsa-miR-335-5p.0.P1.S.36*	3.968634814	8.242992426	34.03440761	28.64254602	5.188789615	4.100893777	77.67116388	58.05551294	284.4183387	tcaagagcaataacgaaaaatga			P1:38.t>a
	hsa-miR-335-5p.3.P0.S.5	3.871838843	13.73832071	52.27431836	16.52454578	4.1869936	2.135882176	44.5177958	42.41456651	332.7186139	tcaagagcaataacgaaaaat		3t:-gt	P0:
	hsa-miR-335-5p.3.P0.S.7	15.14856947	26.10280935	180.7304722	16.96520033	14.89850483	15.33563402	174.3969697	163.8159125	672.9888188	tcaagagcaataacgaaaaatg		3t:-t	P0:
hsa-miR-337-3p	*hsa-miR-337-3p.5.P0.S.1*	49.02715935	8.242992426	8.228098542	28.71598845	23.01562126	2.135882176	311.7954642	547.8471502	98.91836543	tcctatatgatgcctttcttc	5t:-c		P0:
hsa-miR-337-5p	*hsa-miR-337-5p.3.P0.S.1*	9.87318905	13.73832071	5.178523558	11.75078811	8.784980437	0.299023505	253.2643891	315.3950846	34.01954367	gaacggcttcatacaggagtta		3n:+a	P0:
hsa-miR-379-5p	*hsa-miR-379-5p.3.P0.S.2*	31.74907851	10.99065657	7.249932981	18.06683672	18.54606981	1.281529305	408.6067169	888.2217463	26.54272088	tggtagactatggaacgtagga		3n:+a	P0:
hsa-miR-409-3p	*hsa-miR-409-3p.5.P0.S.1*	59.09394034	4.121496213	7.307472132	51.92379496	36.01328238	3.887305559	160.1273499	564.914183	80.74968607	cgaatgttgctcggtgaacccct	5t:+c		P0:
hsa-miR-4284	*hsa-miR-4284.53.P0.S.12*	20.85953177	108.5327336	5.638836763	2.056387919	3.287945895	27.38200949	32.98247443	2.162130831	20.18742152	ggctcacatcaccccata	5t:-g	3n:+a	P0:
hsa-miR-451a	*hsa-miR-451a.0.P1.S.36*	22.45666529	3978.617678	3.279731587	430.5929418	1.643972947	77.31893475	11.44987454	9.706587346	183.3316947	aaaccgttaccaatactgagtt			P1:29.t>a
	hsa-miR-451a.0.P1.S.38	56.04486726	370.9346592	9.292572829	58.46017085	2.209088648	133.0227419	32.81158078	11.68470704	56.6743167	aaaccgttaccagtactgagtt			P1:29.t>g
	hsa-miR-451a.0.P1.S.47	158.0678208	75.56076391	17.77959755	20.34355192	3.159510508	70.31324122	17.77293959	1.472089076	10.76662481	aaaccgttaccattattgagtt			P1:32.c>t
	hsa-miR-451a.0.P1.S.64	359.5970326	90.67291669	37.40044792	5.28785465	10.71151123	52.41454859	27.51387763	4.002242176	6.803908733	aaaccgttaccattactgagtc			P1:38.t>c
	hsa-miR-451a.3.P0.S.12	94.7148577	170.3551768	10.15566009	29.00975815	3.159510508	41.820573	9.740938044	3.496211556	17.04715595	aaaccgttaccattactgagttg		3n:+g	P0:
	hsa-miR-451a.3.P0.S.21	196.6894132	153.869192	3.538657765	14.54160029	6.241959784	57.96784224	12.38978962	2.070125263	12.03768468	aaaccgttaccattactgagtttaga		3n:+taga	P0:
	hsa-miR-451a.3.P0.S.3	432.1940109	210.1963069	38.66630923	35.69301889	10.94269493	156.4320105	29.65004826	5.106308983	21.08464025	aaaccgttaccattactgagtta		3n:+a	P0:
	hsa-miR-451a.3.P0.S.48	219.5332624	181.3458334	19.9373157	14.10094573	7.8088715	71.7656411	52.72069099	16.88302159	15.32748671	aaaccgttaccattactg		3t:-agtt	P0:
	hsa-miR-451a.3.P0.S.49	3092.53448	2843.832387	146.1206731	221.0617013	50.32098443	933.8931224	217.3767227	101.3901351	216.4540196	aaaccgttaccattactga		3t:-gtt	P0:
	hsa-miR-451a.3.P0.S.50	2966.65132	1215.841383	187.8940964	201.3791312	89.77633517	863.1527048	288.8102683	21.3452916	108.2643939	aaaccgttaccattactgagtttag		3t:+tag	P0:
	hsa-miR-451a.3.P0.S.55	2816.133585	1494.729293	58.92009026	247.8681867	82.32708275	615.0913489	239.8492376	32.38595967	58.76782708	aaaccgttaccattactgagtttagt		3t:+tagt	P0:
	hsa-miR-451a.3.P0.S.56	2283.174968	795.4487691	166.6621498	110.8980628	62.05997876	629.2308889	184.7360355	19.73519418	91.29200619	aaaccgttaccattactgagttta		3t:+ta	P0:
	hsa-miR-451a.3.P0.S.57	206974.082	88869.07518	16923.90407	26020.72486	4641.218188	84019.23986	19148.37713	6102.913287	11137.17615	aaaccgttaccattactgagt		3t:-t	P0:
	hsa-miR-451a.3.P0.S.58	23506.80478	8369.384977	3079.092568	1522.534927	710.4788711	7852.827124	2091.909168	242.9407003	1243.76947	aaaccgttaccattactgagttt		3t:+t	P0:
	hsa-miR-451a.3.P0.S.59	32826.32088	30786.20288	2572.374038	3697.091709	740.8409965	18009.16046	3122.483324	1297.784529	2704.366801	aaaccgttaccattactgag		3t:-tt	P0:
	hsa-miR-451a.3.P0.S.8	124.2860269	153.869192	16.54250581	19.31535796	5.342912079	36.2245617	13.67149199	2.438147532	14.72934088	aaaccgttaccattactgagttc		3n:+c	P0:
	hsa-miR-451a.3.P1.S.316	776.7392699	425.887942	95.74514667	88.93877751	22.73306341	364.637805	106.2104034	24.47348089	51.96391835	aaaccgttatcattactgagt		3t:-t	P1:26.c>t
	hsa-miR-451a.3.P1.S.322	198.5285367	137.3832071	17.20420604	38.63071591	2.876952658	71.55205288	15.72221579	4.922297848	8.673114429	aaaccgttactattactgagt		3t:-t	P1:27.c>t
	hsa-miR-451a.3.P1.S.330	28.21602557	5096.916983	2.905727108	532.1638166	1.33572802	35.07118532	10.08272534	3.220194854	107.1428705	aaaccgttaccaatactgagt		3t:-t	P1:29.t>a
	hsa-miR-451a.3.P1.S.332	52.80220222	718.5141731	4.545592901	98.85350498	1.541224638	32.16638556	10.59540629	2.760167018	37.6084186	aaaccgttaccactactgagt		3t:-t	P1:29.t>c
	hsa-miR-451a.3.P1.S.334	53.72176395	644.3272413	6.415615297	143.2861725	2.054966184	85.43528702	31.95711253	4.646281146	86.05823024	aaaccgttaccagtactgagt		3t:-t	P1:29.t>g
	hsa-miR-451a.3.P1.S.378	422.6596077	125.0187185	27.82017934	27.68779449	14.12789252	101.7107092	33.40970855	12.19073766	15.62655962	aaaccgttaccattactgaga		3t:-t	P1:37.t>a
	hsa-miR-451a.3.P1.S.380	614.6060184	427.2617741	45.34085071	53.61297075	15.8489267	150.622411	65.19592743	14.53687963	20.33695797	aaaccgttaccattactgagc		3t:-t	P1:37.t>c
	hsa-miR-451a.3.P1.S.382	116.6391452	42.5887942	10.75982117	18.06683672	3.6218779	39.04392617	16.83302451	5.336322901	5.757153544	aaaccgttaccattactgagg		3t:-t	P1:37.t>g
	hsa-miR-451a.3.P1.S.425	17.37487681	166.2336806	6.012841242	16.89175791	1.001796015	22.25589227	10.50995947	2.070125263	20.71079911	aaaccgttaccagtactgag		3t:-tt	P1:29.t>g
	hsa-miR-451a.3.P1.S.46	157.148259	397.0374685	11.76675631	8.666206232	4.752109301	53.05531324	37.59660298	8.510514971	28.11285367	aaaccgttaccattactc		3t:-agtt	P1:34.g>c
